# Mapping National Plant Biodiversity Patterns in South Korea with the MARS Species Distribution Model

**DOI:** 10.1371/journal.pone.0149511

**Published:** 2016-03-01

**Authors:** Hyeyeong Choe, James H. Thorne, Changwan Seo

**Affiliations:** 1 Geography Graduate Group, University of California Davis, Davis, California, United States of America; 2 Department of Environmental Science and Policy, University of California Davis, Davis, California, United States of America; 3 Division of Ecosystem Services & Research Planning, National Institute of Ecology, Seocheon-gun, Choongnam, South Korea; Fondazione Edmund Mach, Research and Innovation Centre, ITALY

## Abstract

Accurate information on the distribution of existing species is crucial to assess regional biodiversity. However, data inventories are insufficient in many areas. We examine the ability of Multivariate Adaptive Regression Splines (MARS) multi-response species distribution model to overcome species’ data limitations and portray plant species distribution patterns for 199 South Korean plant species. The study models species with two or more observations, examines their contribution to national patterns of species richness, provides a sensitivity analysis of different range threshold cutoff approaches for modeling species’ ranges, and presents considerations for species modeling at fine spatial resolution. We ran MARS models for each species and tested four threshold methods to transform occurrence probabilities into presence or absence range maps. Modeled occurrence probabilities were extracted at each species’ presence points, and the mean, median, and one standard deviation (SD) calculated to define data-driven thresholds. A maximum sum of sensitivity and specificity threshold was also calculated, and the range maps from the four cutoffs were tested using independent plant survey data. The single SD values were the best threshold tested for minimizing omission errors and limiting species ranges to areas where the associated occurrence data were correctly classed. Eight individual species range maps for rare plant species were identified that are potentially affected by resampling predictor variables to fine spatial scales. We portray spatial patterns of high species richness by assessing the combined range maps from three classes of species: all species, endangered and endemic species, and range-size rarity of all species, which could be used in conservation planning for South Korea. The MARS model is promising for addressing the common problem of few species occurrence records. However, projected species ranges are highly dependent on the threshold and scale criteria, which should be assessed on a per-project basis.

## Introduction

Species distribution model (SDM) applications use the climate and occasionally the environmental characteristics of known species occurrence locations or abundance to estimate a likelihood of occurrence at other locations where no occurrence information is available [1]. Multiple SDMs can be used to identify national patterns of high biodiversity for conservation network planning [2]. However, the number of occurrence records available per species to develop a model varies from few to many. Some species have rarely been recorded or electronic data are not available, which makes them difficult to model using a SDM because model quality is influenced largely by the number of records used in model building [3,4]. In addition, there may remain undiscovered species, some regions have sparse ecological surveys, and we cannot be assured of the data quality for each species.

Multi-response SDMs may offer benefits for applications with large numbers of species that include species with few records ([5]; ‘Multi-response’ models were previously called ‘community’ models). Multi-response SDMs combine all species data and use information on the presence of other species to supplement information for the modeled species. The potential benefits of a multi-response SDM include a possibly more accurate representation of biodiversity, and a method to synthesize complex data into a simpler form. This method may be useful for modeling rarely recorded species of conservation concern which otherwise would be excluded from regional analyses, because significant predictor variables for some species identified by the MARS algorithm may help to inform how species with few presence points react to the same predictor variables. Multi-response SDMs produce information on spatial patterns of biodiversity at a multi-species level as well as at an individual level [5–7].

Accurate information on the distribution patterns of as many species as possible is crucial for attempts to assess regional biodiversity variations and develop conservation plans; however data inventories are insufficient in many areas [8]. This is especially true for publically available records for vascular plants in South Korea, which total 2,012 points comprising 289 species. Many species have only one or few occurrence points, which makes utilizing an individual-level SDM and validating a model almost impossible.

We addressed these challenges by using the Multivariate Adaptive Regression Splines (MARS) multi-response SDM model which can be used to simultaneously model multiple species that have varying numbers of observation records [9]. We conducted a sensitivity analysis on four methods of selecting range projection thresholds from the MARS outputs, to see which one could be the most useful for assessing patterns of biodiversity.

The MARS algorithm considers interactions between predictor variables locally, and is computationally efficient since each species does not have to be modeled individually [4,6,10]. MARS SDMs have been tested in various ways. The MARS multi-response model performed particularly well among other methods for predicting occurrence patterns of species in a study that compared SDM modelling methods, including individual-level SDMs using a large international dataset [6]. Mateo *et al*. [4] found that optimal minimum occurrence points were 18–20 for running MARS; Elith and Leathwick [11] tested using presence-only data in the MARS modeling; and Leathwick *et al*. [12] used the MARS model to predict the distribution probabilities of freshwater diadromous fish.

The goals of this study are to test the suitability of the MARS model to spatially portray rare plant species ranges for the development of maps describing national plant species richness patterns. Because we are working with species that have low numbers of recorded observations, we used the MARS algorithm. We test the suitability of the SDM outputs to produce as much spatial resolution as the data permit through comparison of alternative range cutoffs, the use of an independent species occurrence data set to test predicted range map accuracy, and a spatial examination of potential pseudoreplicate values in predictor variables to rank the confidence we have in the rare species ranges produced.

## Materials and Methods

There were five main steps to the analysis: 1) Gather vascular plant species occurrence records and climate and environmental data for South Korea; 2) Run MARS models and evaluate the outputs; 3) Develop occurrence probability maps for each species; 4) Assess the spatial effect of the use of different threshold values for the development of range maps; and 5) Identify the spatial patterns of high plant biodiversity areas.

### Study area

**Fig 1:**
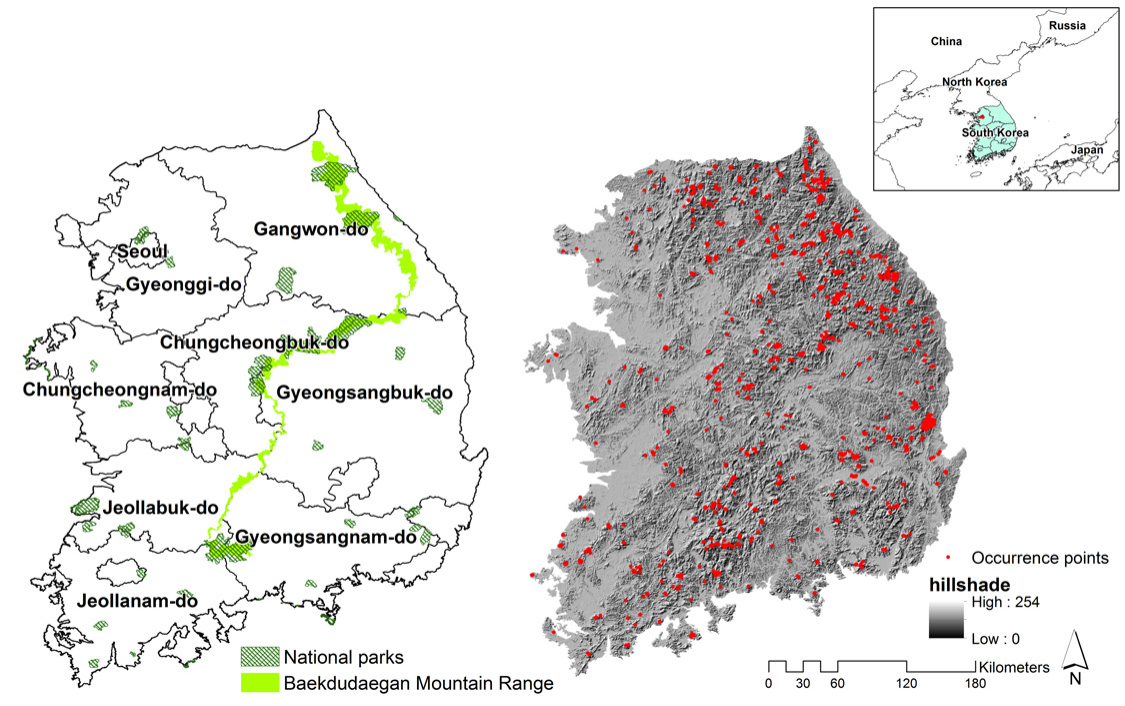
National parks and high biodiversity mountain range (Baekdudaegan mountain range) (left), and locations of modeled occurrence points and topography of South Korea (right).

South Korea constitutes the southern Korean Peninsula (Fig 1). South Korea's total land area is 100,033 km^2^, and it has a population of 48.0 million people [13]. The Korean Peninsula is topographically complex, with mountains covering around 70% of the land. The primary mountain range stretches along the east coast of South Korea. The mountains interface with southern and western coastal plains that produce most of the agricultural crops of South Korea. This study was confined to mainland of South Korea (95,219 km^2^), and excludes its large number of islands.

### Species data

The publically available national survey data for vascular plants in South Korea consists of 2,012 points, representing 289 species (The Second National Ecosystem Survey data; http://library.me.go.kr/search/Search.Result.ax?sid=21&s=CID&st=DESC; S1 Dataset). Each point represents the occurrence of a single species [14]. The South Korean Wildlife Protection and Management Act (enacted in 2004, amended in 2014) has designated 77 vascular plant species as endangered species, of which 18 are identified by 68 points in the national survey data. In addition, 50 endemic species are identified by another 307 points in the survey data. The overall number of individual species occurrence records varies from one to 69, and consist only of presence points. We used another independent national survey data (The Third National Ecosystem Survey data) to test the probability cutoff method options for 166 species found in both datasets and to define each species’ range (S1 Dataset).

### Environmental variables

We used a series of climate and topographic variables as predictors in the species modeling. Climatic variables are important factors that influence species’ distribution particularly for plants [15]. Bioclim is a series of 19 variables derived from monthly temperature and rainfall values as potentially ecologically meaningful predictors [15,16]. Bioclim varliables were originally developed for species distribution modeling and have been widely used [10,17]. We obtained 30 arc-second resolution (~ 1 km) Bioclim variables for current and future times from the WorldClim website ([16]; http://www.worldclim.org/).

Topography can influence local climate conditions with resulting effects on plants by varying sunlight, soil moisture, and nutrients [18]. Topographic data (30 m resolution) were obtained from the Korean Water Management Information System (WAMIS), and we calculated aspect and northness (the sine of aspect) for each species location [19].

A subset of the predictor variables was selected by running a principal components analysis of the 23 potential predictor variables across the study area and selecting 11 of the least correlated predictors from among Bioclim variables [20,21]: annual mean temperature, temperature seasonality, mean temperature of the warmest and coldest quarter of the year, annual precipitation, precipitation of the wettest and driest quarter of the year; and the topographic variables: elevation, slope, curvature, northness, to build the models. The selected predictor variables were used in the subsequent modeling.

We used environmental variables from different data sources as predictors in the species modeling. Matching raster objects (in terms of origin and resolution) is required to conduct spatial analysis in R using the ‘raster’ package. Because the climate and environmental variable rasters we used have different data structures (grid cell sizes), we needed to decide on the operational grid scale for the modeling, either 30 m or 1 km.

To make this decision, we conducted two analyses. First, we resampled the 30 m elevation raster to 1 km grid scale by using bilinear interpolation, and checked the elevation variations on each species’ occurrence points between 30 m and 1 km elevations. Second, we calculated the distances between points for each species to check any significant problems from possible raster replicates that could originate in the resampling of the Bioclim variables to 30 m.

### Modeling technique

MARS is a generalization of stepwise linear regression, and is appropriate to large numbers of predictor variables as a modification of the regression tree approach. Knots, points where the coefficients of predictor variables change along the extent of each predictor variable, are used to select relevant value ranges for each predictor, and a linear response is modeled for each section (S1 Fig) [4,6,10].

We used the ‘extract’ and ‘predict’ functions in the ‘raster’ package [22] found in R (version 3.0.2) to extract environmental values from each occurrence point and to predict occurrence probability of each species in study area, and the ‘mda’ package [23] to run the MARS model.

MARS fits interactions between predictors automatically and selects predictor variables by using the signal from many species simultaneously [11]. To output suitability surfaces for each species, we re-scaled the predictor variable grids selected by MARS to 0.1 km^2^ rasters to lower computational demands. We used Elith and Leathwick’s codes [11] to constrain predictions between 0 and 1. MARS originally only accommodates normal error terms as its available algorithms, so Elith and Leathwick [11] reprogrammed MARS to be able to run a generalized linear model (GLM) with a binomial error distribution.

We assumed that species with many occurrence points would contribute to better overall model outputs. However, we used all species with at least two observations in modeling because this provided outputs for the highest number of species, including endangered and endemic species that would otherwise have been excluded. Thus, the total number of species modeled was 199 out of 289 species, including 12 endangered species and 35 endemic species, two of which are endangered (S1 Table).

The independent survey data shared 166 species with the modeled data and the number of points is 7,352 (S1 Table). We used the independent data in a sensitivity analysis to select the most appropriate cutoff value for defining the presence areas, or range map, of each species.

### Choice of threshold to find species range

A threshold (or cut-off) is required to transform occurrence probabilities from the models into binary presence or absence values that can represent potential species’ ranges. However, the selection of thresholds is one of the least investigated areas in the SDM studies [24]. The MARS software combines all species data and supplements information from the presence of more common species to help select the relevant predictor variables and determine the occurrence likelihood for the rarer species in a dataset. This was the case for many species in our data, whose records are few and consist of only presence records. Therefore, an alternative approach from commonly used techniques to define range cutoffs (e.g. assessment of sensitivity and specificity based on varying the probability thresholds; [10]) was needed.

Nenzen and Araujo [24] identified three categories of threshold optimization methods: 1) fixed, 2) data-driven (using species data and predicted probability values), and 3) accuracy-based (using the threshold in the best agreement between the evaluation and the original data). For each species’ cutoff values, we extracted the modeled occurrence probabilities from the location records of each species separately using the data-driven approach, and evaluated them in two ways. For each species, we took the mean, median, and one standard deviation value of the occurrence probabilities from each species’ occurrence points, and used these three values as cutoffs to develop range maps from probability maps, in a sensitivity analysis to select the most appropriate cutoff value. For the mean and median values, we identified as range all map areas with equal or greater probability as part of each species’ range. For one standard deviation cutoff, we classified map areas with values within one standard deviation as the presence areas. The standard deviation thresholds include the values to either side of the mean, and therefore render as range some areas with probabilities lower than the mean value. The representation of occurrence areas of species can be wrong in two ways: false presence and false absence [25]. Since we did not have absence data to be used for balancing threshold values, we wanted to define areas where the underlying data were well reflected. Thus, we devised the inside areas of one standard deviation as threshold to reduce the risk of false presence and false absence errors.

According to Liu *et al*. [26], max SSS threshold (maximizing the sum of sensitivity and specificity) is promising when using presence only data, and it is not affected by pseudo-absences. Therefore, we calculated the max SSS threshold value for each species using inventory pseudo-absences, which are the locations of concurrently modeled species used by MARS model to fit a model for each species, and generated output probability values. We then compared our data-driven threshold values to the max SSS threshold values which are an accuracy-based approach to generating thresholds. We generated range maps and calculated the percentage of each species’ occurrence points from the independent survey data occurring in the corresponding range map derived from each of the four threshold cutoff approaches.

Because many species have a few occurrence records and the number of species’ point data varies, we examined whether the number of species’ occurrence points influences model results and range maps derived from each threshold, by iteratively excluding modeled species with <3, <5, <7, and <10 records in the original species location records.

As a second check on the selection of appropriate threshold level for production of range maps, we combined the binary range maps of all species under each of the four cutoff options and assessed the spatial patterns of national-scale high species richness areas derived from each cutoff method to see if they identify the Baekdudaegan Mountain Range, which is a known high biodiversity area [27]. We used these two lines of evidence to select the most appropriate threshold method.

### Identification of high plant biodiversity areas

The binary range maps of each species were combined to portray patterns of plant biodiversity on a 0.1 km^2^ basis. We categorized the species richness maps into three groups: 1) all species, 2) endangered and endemic species, and 3) range-size rarity weighted maps from all species.

Endemism refers to a species being limited to a particular area regardless of the size of that area, while range-size rarity is the reciprocal of the calculated range size, which weights restricted species more heavily when summing species richness values [28]. Hotspots of range-size rarity are important because these areas are richest in the most restrictedly distributed species [29]. Range-size rarity was calculated for all the species in a grid cell as the sum of each species’ inverse range extent in our study. Then, we computed a correlation matrix between each combination of two maps from among the three species richness maps to find dependencies among these groups.

In South Korea, the Baekdudaegan Mountain Range is a known high biodiversity area (Fig 1) [27]. We overlaid the maps of Baekdudaegan Mountain Range as well as all national parks with our various maps of species richness, to assess the accuracy of the species richness maps.

## Results

### Resolution of environmental variables

We found elevation differences between the two resolution (30 m and 1 km) elevation rasters for each species (S2A Table). The range of differences of each species’ average elevation is from -117 to +221 m. The minimum and mean distance between occurrence points for all species is 25.4 and 92.6 km, respectively, beyond the 1 km at which values from the Bioclim variables would be the same for two or more points (S2B Table). For two species the mean distances between points are less than 1 km.

Therefore, we resampled the 1 km Bioclim variables to 30 m resolution to create input values for the MARS modeling by using bilinear interpolation. We selected the 30 m grid for model operational resolution, to retain the more detailed topographic information because of South Korea’s complex topography.

### Fitted MARS model

A small set of predictors plays a dominant role in explaining the probability of occurrence for the plant species in South Korea. Predictor environmental variables selected by the model were precipitation of wettest quarter, mean temperature of coldest quarter, elevation, and mean temperature of warmest quarter. Examination of the marginal contribution of each predictor variable in the model shows that precipitation of wettest quarter had the most explanatory power among these variables (Table 1). Changes in residual deviance were calculated while dropping each predictor variable in the final model and averaged across all species [12].

**Table 1:** Environmental variables used in the model.

	Minimum value	Maximum value	Knot value	Delta deviance	Rank
Precipitation of wettest quarter^1^	450	1034	707	4.72	1
Mean temperature of coldest quarter^2^	-109	43	-3	0.62	2
Elevation^3^	1	1895.27	1^st^: 307.97 2^nd^: 830.34	0.49	3
Mean temperature of warmest quarter^4^	144	253	242.02	0.00	4

1. Unit: Milimeters

2. Scaling Factor: 10, Unit: Degrees Celsius

3. Unit: Meters

4. Scaling Factor: 10, Unit: Degrees Celsius

The reduction in deviance for each species with a null model ranges from 2.08 to 191.94, and the pseudo R-squared that represents the proportion of the total deviance explained by the model ranges from 0.02 to 0.90. On average, the model accounted for 20% of the null or total deviance [30].

### Cutoff thresholds for binary selection

We used the presence points from each species in the independent survey data as a way to check the performance of range maps produced from different cutoffs. The average percent of points inside presence areas from each threshold value was: 20% under the mean threshold, 22% under the median threshold, 43% within one standard deviation, and 38% under the max SSS threshold (Table 2). Concerning the number of species’ occurrence points, the number of records available influenced the performance of model and a range map from all four cutoff options (Table 2). The higher the number of occurrence points, the higher proportion of independent survey data that was included in the range maps derived from cutoff values.

**Table 2:** Proportion of the occurrence points from the independent survey data included in the presence areas by using each cutoff threshold and analysis results of species richness maps using each cutoff.

	Proportion of occurrence points included in species range maps for								Analysis of total species richness maps		
Cutoff threshold approaches	All species	Common species	Endangered species	Endemic species	Species with ≥ 3 records	Species with ≥ 5 records	Species with ≥ 7 records	Species with ≥ 10 records	Maximum number of species	Area (km^2^) containing > 100 species	Area (km^2^) containing > 50 species
Mean	0.20	0.20	0.32	0.20	0.21	0.25	0.27	0.27	118	19	1,962
Median	0.22	0.22	0.35	0.22	0.24	0.27	0.30	0.31	118	19	2,371
1 SD	0.43	0.42	0.44	0.50	0.44	0.50	0.54	0.57	107	2,542	31,927
Max SSS	0.38	0.37	0.59	0.40	0.41	0.46	0.51	0.54	120	19	15,797

**Fig 2:**
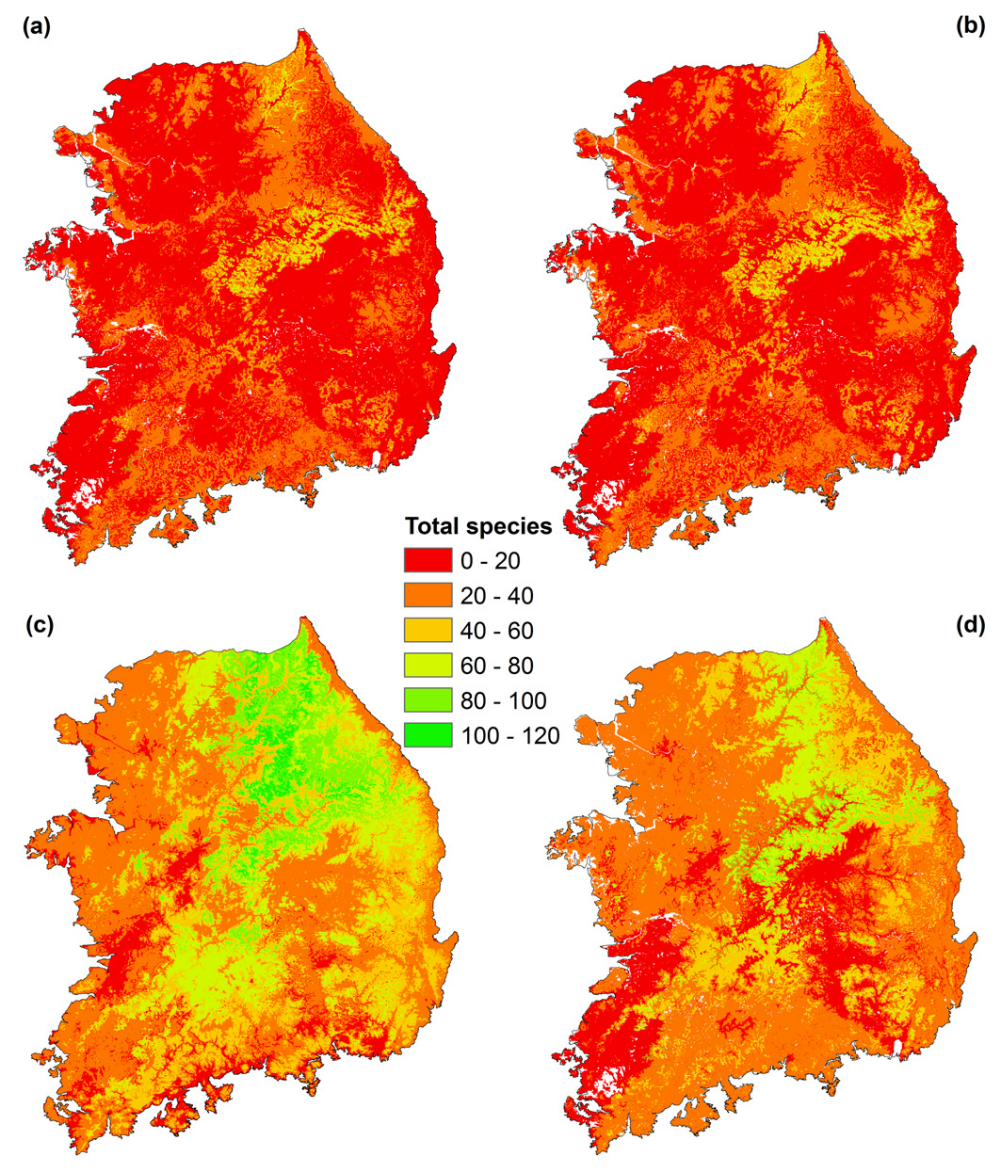
Total species richness from each cutoff threshold: (a) Mean, (b) Median, (c) 1 SD, and (d) Max SSS.

Species range extents were greatly affected by the selection of thresholds, and also affected total estimated species richness values that ranged, depending on the threshold chosen, from 107 to 120 (Table 2 and Fig 2). When using ‘mean’ as the cutoff, areas with potential for more than 100 species were 0.02% (19 km^2^) of the study area, and for more than 50 species were 2.06% (1,962 km^2^) of the mainland of South Korea. When using ‘median’ as the cutoff, areas have potential for more than 100 species were 0.02% (19 km^2^) of the study area, and for more than 50 species were 2.49% (2,371 km^2^) of the mainland of South Korea. By using one standard deviation as the cutoff, areas have potential for more than 100 species were 2.67% (2,542 km^2^) of the study area, and for more than 50 species were 33.53% (31,927 km^2^) of the mainland of South Korea, which were the highest among the four cutoff options. In the case of using ‘max SSS’ as the cutoff, areas have potential for more than 100 species were 0.02% (19 km^2^) of the study area, and for more than 50 species were 16.59% (15,797 km^2^) of the mainland of South Korea. All combined species richness maps from the four cutoff methods showed that the central part of South Korea (the boundary between Chungcheongbuk-do and Gyeongsangbuk-do) has high species richness. However, we found that the Baekdudaegan Mountain Range, which is a known high biodiversity area [27], was somewhat underestimated in the combined range maps from mean, median, and max SSS cutoffs.

Since the range maps derived from one SD cutoffs performed better than those from max SSS cutoffs in the cross-validation with the independent survey data and the Baekdudaegan Mountain Range was evaluated as high species richness areas in the combined range map from one SD cutoffs, we conclude that the data-driven cutoff approach is appropriate in our study. Thus, we selected the within one standard deviation threshold for systematic use in the following analyses, to minimize omission and commission errors.

### High biodiversity areas

**Fig 3:**
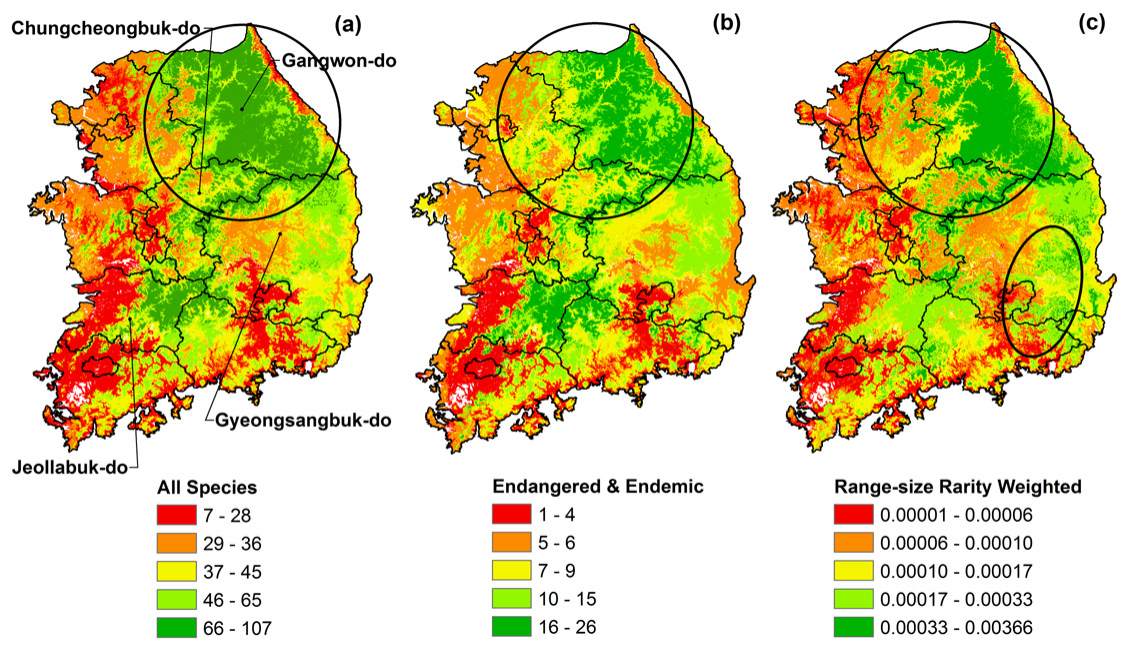
**Species richness maps from each group category, using the one standard deviation thresholds: (a) All species, (b) Endangered and endemic species, (c) Range-size rarity weighted maps from all species.** Non-zero values are grouped into five classes, each class contains equal numbers of grid cells (quintile).

When we combined the binary range maps of all species using the one standard deviation threshold values, all of the cells with the highest number of species has an estimated 107 species (1.33 km^2^) and all of the cells with the highest number of endangered and endemic species has an estimated 26 species (118 km^2^) (Fig 3A and 3B).

The model predicts the highest species richness in Gangwon-do province (the central & eastern northern South Korea, in the circle of Fig 3A) and around central South Korea (on the border between Chungcheongbuk-do province and Gyeongsangbuk-do province, and the eastern part of Jeollabuk-do province). Endangered and endemic species richness is also highest on the middle part of Gangwon-do province, but not across the entire area (in the circle of Fig 3B). These are also generally high on the border between Chungcheongbuk-do province and Gyeongsangbuk-do province, and extending to the eastern part of Jeollabuk-do province (Fig 3B). High species richness areas near Gangwon-do province are broadly the same areas that are rich in species with restricted ranges within South Korea (in the circle of Fig 3C). However, highly-ranked areas of range-size rarity are also distributed along the east coast of South Korea (in the oval of Fig 3C) and are low in Jeollabuk-do province, which differs from total species richness.

Pairwise correlation of total species richness and endangered and endemic species richness is moderately correlated (0.58). Species richness for range-size rarity is also moderately correlated with species richness for endangered and endemic species (0.61), and has a high correlation with total species richness (0.79).

Based on our combined maps, the Baekdudaegan Mountain Range includes the areas with the highest estimated number of species (107) and the highest number of endangered and endemic species (26). Areas in national parks also include the highest predicted numbers of species (107) and highest number of endangered and endemic species (26). In terms of total species richness, 67.3% of the Baekdudaegan Mountain Range and 44.4% of the national parks present the highest values of predicted richness (66–107 species) (S2 Fig). These areas represent 20% of mainland South Korea. For endangered and endemic species, 56.4% of the Baekdudaegan Mountain Range and 39.9% of national parks are estimated to harbor species richness in the highest quintile of species richness for endangered and endemic species in mainland of South Korea, from 16 to 26 species.

## Discussion

This study identified biodiversity hotspot areas for vascular plants in South Korea by using an inventory that includes many species with too few observations to reliably project a range map with standard SDM techniques. We addressed this weakness by deploying a multi-response SDM to take advantage of occurrence data from more common species that frequently co-occur with the rare species for which we have sparse data. Using this approach, we were able to better predict potential locations of plant biodiversity hotspots in South Korea. Because model quality is influenced by the number of occurrence records used, models of species with few occurrence points likely increase the level of uncertainty in the overall national species richness maps (Table 2). However, our objective was to portray patterns of plant species richness including rare species, which is why the MARS model was used. We found that national species richness maps were more spatially detailed by including the species with five or fewer observation points, and that the spatial patterns of species richness differed when these were included (S3 Fig). Moreover, some of the hotspots predicted using all species correspond well to hotspots identified in the biodiversity report from South Korean Ministry of Environment [27]. However, the hotspot areas we identified may not be representative of all 4,130 plant species in South Korea [31]. This is because the distributions of rarely recorded species are not strongly nested within the distributions of widespread species, as shown by only moderate correlation values between total species richness and endangered and endemic species richness. Therefore, many other plant species with few or no occurrence records could potentially identify other hotspot locations. These results agree with Williams *et al*. [29] that overall species richness does not always provide a reliable surrogate for rare species. Therefore, total species richness cannot be the only criterion for identifying biodiversity hotspots in South Korea. This finding corresponds to previous work suggesting that species richness hotspots are largely driven by common, widespread species, and concentrating on protecting diversity hotspots can miss species most in need of conservation [32]. In the event that these results would be used in a conservation planning exercise for South Korea, the distributions of endangered, endemic and range-size rarity maps should also be considered.

### Rarely recorded data

Previous studies on the effects of sample size on individual-level SDMs have assessed model performance using different modeling methods, different spatial scales, and differing environmental variables [33,34]. In our study, a MARS multi-response model was used to handle a large number of species, including rarely recorded ones, and to develop overall plant biodiversity information for South Korea. One advantage to this approach is that the MARS multi-response SDMs use information on the presence of other species in predicting suitability for rare species. Under this framework, we were able to include 12 endangered and/or endemic species that have only two occurrence points and 32 endangered and/or endemic species with less than 10 data records (26 endangered and/or endemic species with less than 6 data records), which under single species level SDM approaches would likely have been excluded (S1 Table). The inclusion of such species could be critically important in estimating patterns of plant biodiversity for conservation planning, partly because model outputs that include locations for rare species are more useful for conservation practitioners than those that do not. Multi-response SDMs such as MARS are becoming more available. Further tests on the degree to which species with more occurrence records can improve the modeling of less commonly reported species can help the conservation community understand the useful applications of this approach.

However, model quality is largely influenced by the number of records. We found that the higher proportions of the independent occurrence records were included in the modeled range maps for species with more than 10 occurrence records (0.57 using one SD cutoffs) compared to species with less than 9 occurrence records (0.36 using one SD cutoffs) on average (Table 2 and S1 Table). The scarcity of occurrence points for some species also made it difficult to derive alternative threshold statistics. For example, 41 species in the data set had only two location records. Thus, more complete survey data can bring better modeling results. The South Korea national ecosystem survey should be updated to cover as many species as possible including rare species for further regional biodiversity assessments or conservation plan developments.

### Pseudoreplication in predictor variables

There is some risk of pseudoreplication when we resample the environmental variables to smaller grids because a species’ occurrence points may wind up sampling from the same climate variable grid cell if they are too close together. However, given the low number of occurrence records for many species, and the desire to map as many as possible for inclusion in national species richness maps, we retained all species records.

While the average minimum distance between each species’ occurrence points is 25.4 km, which indicates no risk of pseudoreplication, we assessed which species’ models might be subject to pseudoreplication because two or more occurrence points occurred within the extent of the origin-scale Bioclim grid cells (925 m average), as a way to relatively rank confidence in individual species’ models. Fifty three species have duplicate climate values because of two or more occurrence records are within a single grid cell. Overall, 146 species (73.4%) do not have multiple samples from a single grid cell. The degree to which these pseudoreplicates affect model outputs is likely further to depend on the number of occurrence records. For our least well recorded species, less than or equal to five occurrence points, there are eight species that have one double sample each: *Abies koreana*, *Allium senescens*, *Cardamine lyrate*, *Cephalotaxus koreana*, *Cnidium tachiroei*, *Deutzia coreana*, *Forsythia ovata*, *Thymus quinquecostatus* (S2B Table). When considering range models for individual species, maps from these eight should be considered potentially less accurate. However, we feel that the overall effect of this potential pseudoreplication on patterns of plant biodiversity at the national scale is likely minimal. This is due to South Korea’s relatively small area extent and its very complicated topography, with a large number of successive mountain ranges across the entire country (Fig 1). Topography can influence local climate conditions on plants by varying sunlight, soil moisture, and nutrients [18], and retaining as much of this detail in this study was a strategic decision which in this case provided more detail through improved elevational detail, and more species than was potentially lost due to some model inaccuracies.

### Cutoff selection and implications

Among many factors that contribute to uncertainty in SDM results, selection of cutoff values for transforming probabilities of occurrence into binary (presence or absence) range maps is one of the least explored areas [24]. Previous multi-response SDM studies have mainly pioneered the modeling technique itself or been focused on improving reliability by exploring the effects of the number of presences [4,11,12]. However, the effect of different cutoffs in these models has been little investigated.

We used independent presence data from another survey in an exercise to evaluate the performance of maps selected under the different cutoffs. The one SD cutoff produced reasonable accuracy scores when compared to the mean or median cutoffs that have been used in other studies [24]. With the independent survey data, and we found that the one SD cutoff performed better than the max SSS cutoff which has been considered a promising cutoff method when only presence data are available [26].

Specificity could be calculated with pseudo-absences generated by the MARS program during species distribution modeling, but is likely to be inaccurate in our study. Because the large number of pseudo-absences is almost the same for all species (MARS uses all occurrence points of other concurrently modeled species as pseudo-absences for each modeled species), each specificity value depends on modeled range size rather than true negatives. The larger the modeled range, the lower the specificity values will be. Since the one SD threshold produces a larger range size than the mean or median threshold, a systematic result in model performance using this approach can be detected. Average specificity values for all species under mean and median cutoffs were almost perfect (0.90 and 0.89, respectively), and average specificity under one SD was 0.68. Thus, specificity using the pseudo-absences that MARS generates is not an informative test of model performance with our results.

We conclude that restricting predicted range size is beneficial for subsequent analysis. This result is similar to Calabrese *et al*. [35], who demonstrated that stacking thresholding occurrence probabilities would tend to over-predict species richness. The approach used here to develop a conservative view of species range size is similar, and we found confining presence areas can reduce errors from modeling. In conclusion, our study results show that different cutoff values have great influence upon projected spatial patterns of plant biodiversity and the use of data-driven cutoffs according to circumstances and regional context is highly recommended.

Our application of MARS with rarely recorded data for projection of biodiversity hotspots is one of the first in SDM studies. We found it performs well, even considering the paucity of reliable occurrence data for many species. However, data status is still very important in running a SDM model, since species occurrence points are directly related with environmental information where the points are located. Among our occurrence data, observation data were largely collected in mountainous areas. Only 13% of the records are of low elevation or mesic-adapted species. Thus, it is possible that these results are representative of only mountainous species. Therefore, we recommend an effort to more completely collect plant species records, with a focus on areas that have been under-reported.

Biodiversity on the Korean Peninsula is under threat due to habitat loss from urbanization, industrialization, and climate change. Therefore, protection of biodiversity in the major ecological regions and expansion of protected areas is important for conservation [27]. Although the number of species in the data is not enough to represent the 4,130 plant species in South Korea [31], our biodiversity assessment results could be used to prioritize some locations for conservation planning. Our results agree with national assessments [27] that most of the Baekdudaegan Mountain Range has high biodiversity. However, there generally is not enough biodiversity information beyond this area. This study is one of the first to find potentially high-biodiversity areas outside of the Baekdudaegan Mountain Range. Those areas could become candidates for priority biodiversity surveys, and potentially for addition to the national network of national parks and protected areas. Encouragingly, the current extent of protected areas overlaid with our model outputs showed that some of the most species rich areas are already under conservation management.

## Supporting Information

S1 Dataset Survey data and independent testing data. The “SurveyData.csv” file includes the plant species’ locations from the South Korea Second National Ecosystem Survey. We used data from another independent national survey (The Third National Ecosystem Survey) to test our method. The “TestData.csv” file includes the 166 plant species’ locations found in both datasets.
(ZIP)

S1 Fig We provide five fitted MARS functions as examples of how the model selects relevant segments of predictor variables for model fitting. (PDF)

S2 Fig The Baekdudaegan Mountain Range and the national parks on the species richness maps of South Korea. (PDF)

S3 Fig Species richness maps by number of occurrence points from each group category, using the one standard deviation thresholds: (a) All species, (b) Endangered and endemic species, (c) Range-size rarity weighted maps from all species.
(PDF)

S1 Table The list of modeled plant species. (PDF)

S2 Table Elevation variations on each species occurrence points between 30 m and 1 km elevation rasters, and distance between points for each species. (PDF)
