# Supplementary material for: Mapping National Plant Biodiversity Patterns in South Korea with the MARS Species Distribution Model
Source: PLoS One. 2016 Mar 1;11(3):e0149511. doi: 10.1371/journal.pone.0149511 (PMC4773094; doi:10.1371/journal.pone.0149511)
Supplement: S1 Fig — (PDF) [file pone.0149511.s002.pdf]

S1 Fig. Five fitted MARS functions as examples.

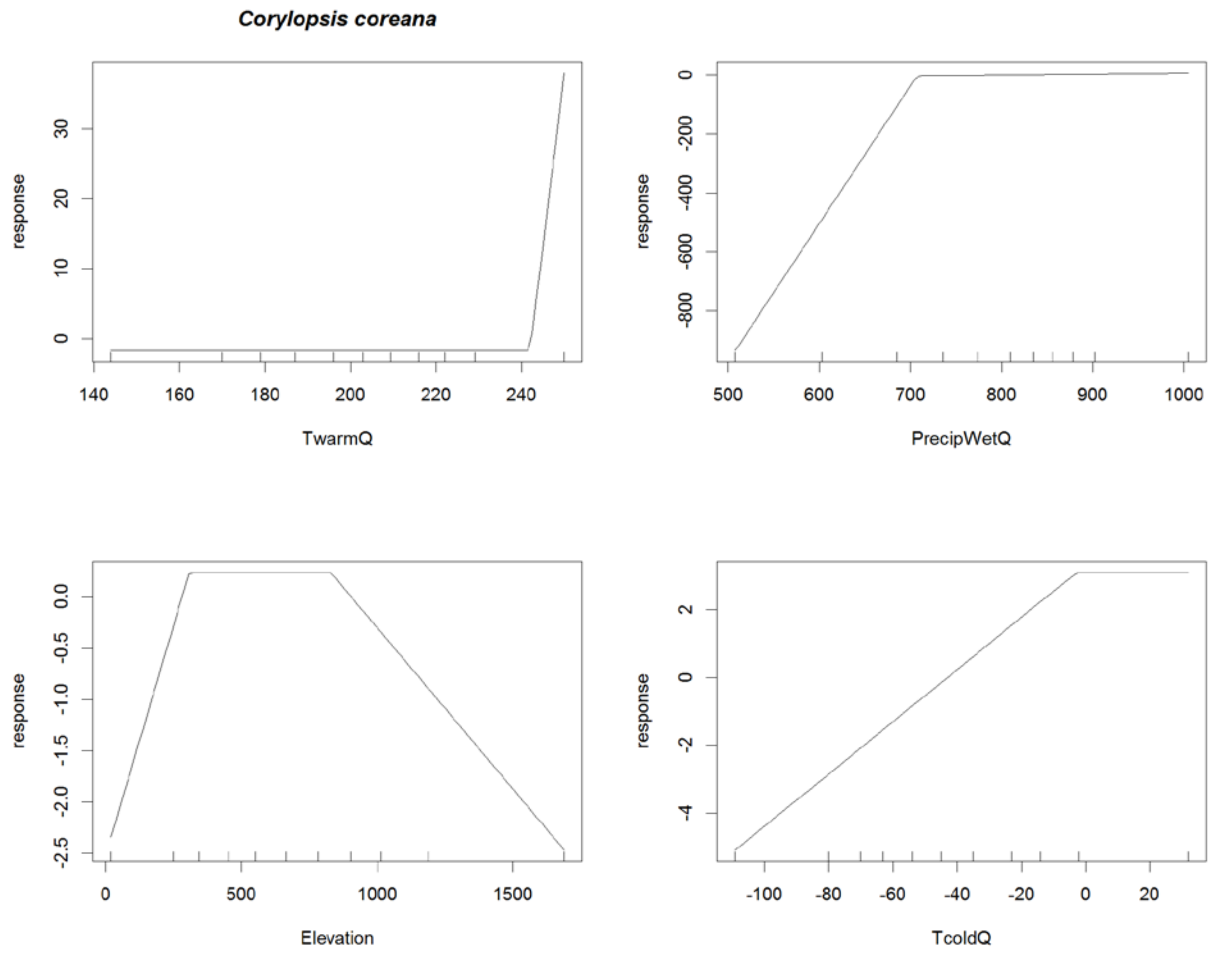

S1 Fig 1. Fitted MARS functions for *Corylopsis coreana* (Hamamelidaceae) (23 occurrence records). (TwarmQ: Mean temperature of warmest quarter, PrecipWetQ: Precipitation of wettest quarter, TcoldQ: Mean temperature of coldest quarter)

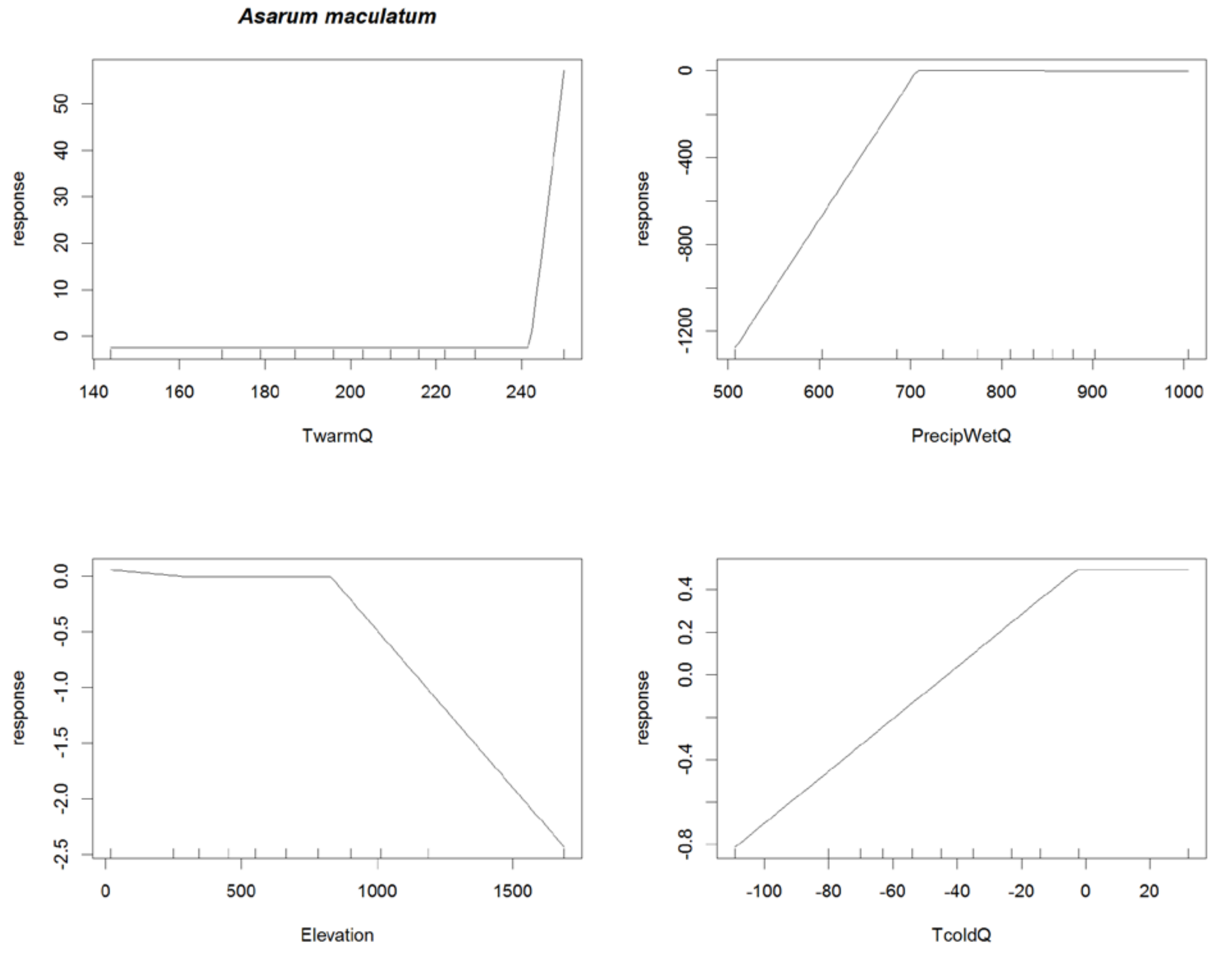

S1 Fig 2. Fitted MARS functions for *Asarum maculatum* (Aristolochiaceae): South Korean endemic species (2 occurrence records). (TwarmQ: Mean temperature of warmest quarter, PrecipWetQ: Precipitation of wettest quarter, TcoldQ: Mean temperature of coldest quarter)

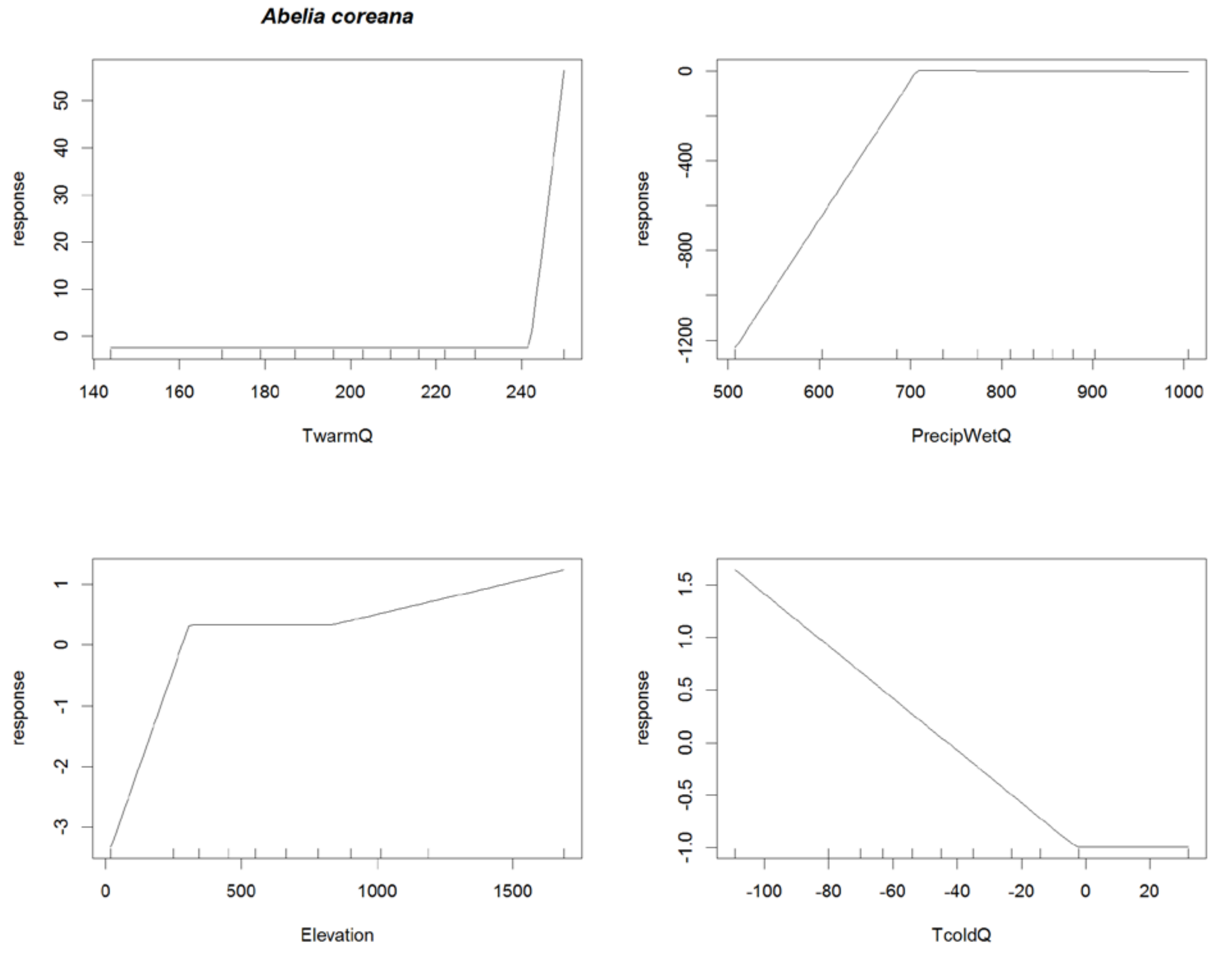

S1 Fig 3. Fitted MARS functions for *Abelia coreana* (Hair Abelia) (7 occurrence records).

(TwarmQ: Mean temperature of warmest quarter, PrecipWetQ: Precipitation of wettest quarter, TcoldQ: Mean temperature of coldest quarter)

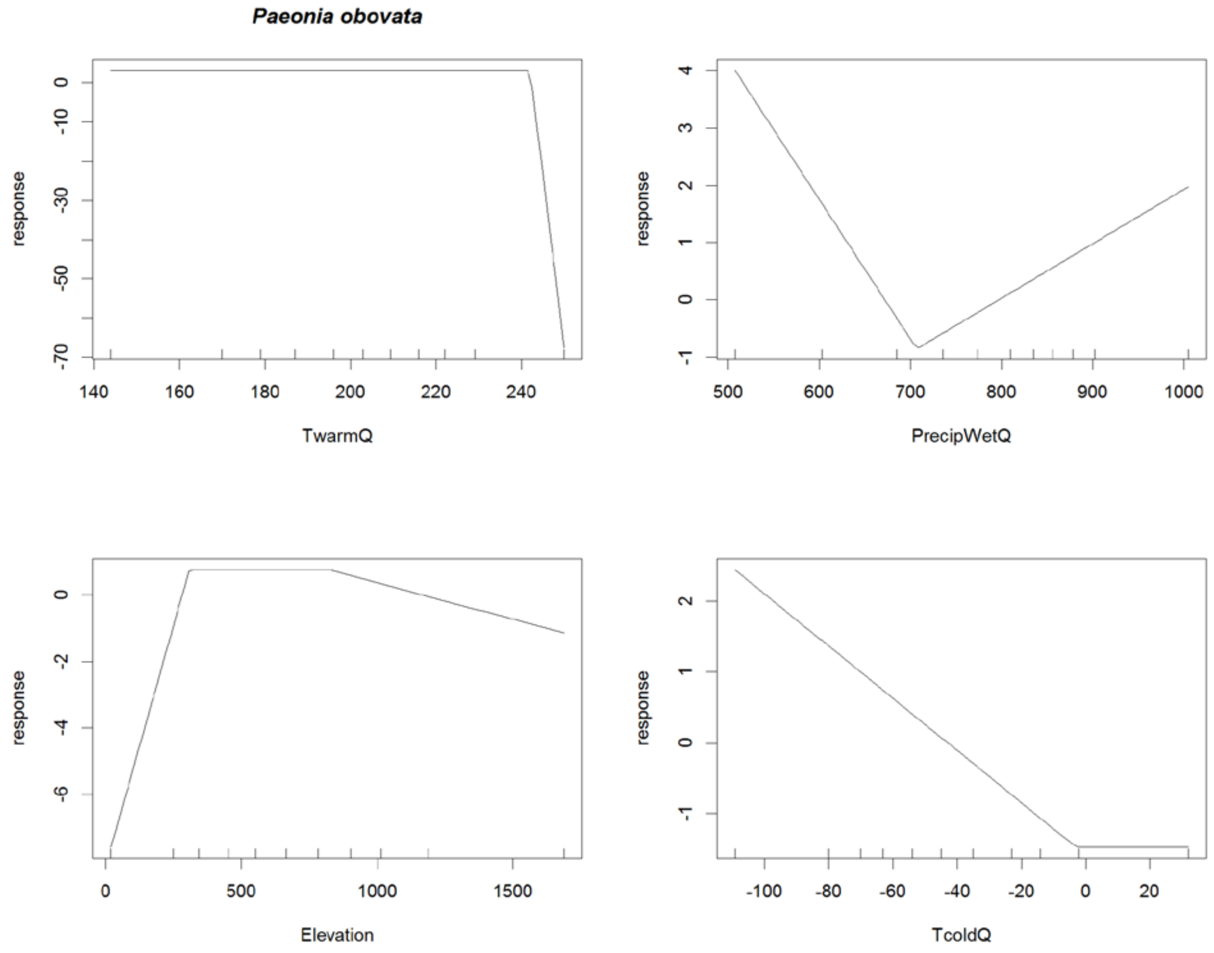

S1 Fig 4. Fitted MARS functions for *Paeonia obovata* (Woodland Peony): Endangered Species Class Ⅱ (17 occurrence records). (TwarmQ: Mean temperature of warmest quarter, PrecipWetQ: Precipitation of wettest quarter, TcoldQ: Mean temperature of coldest quarter)

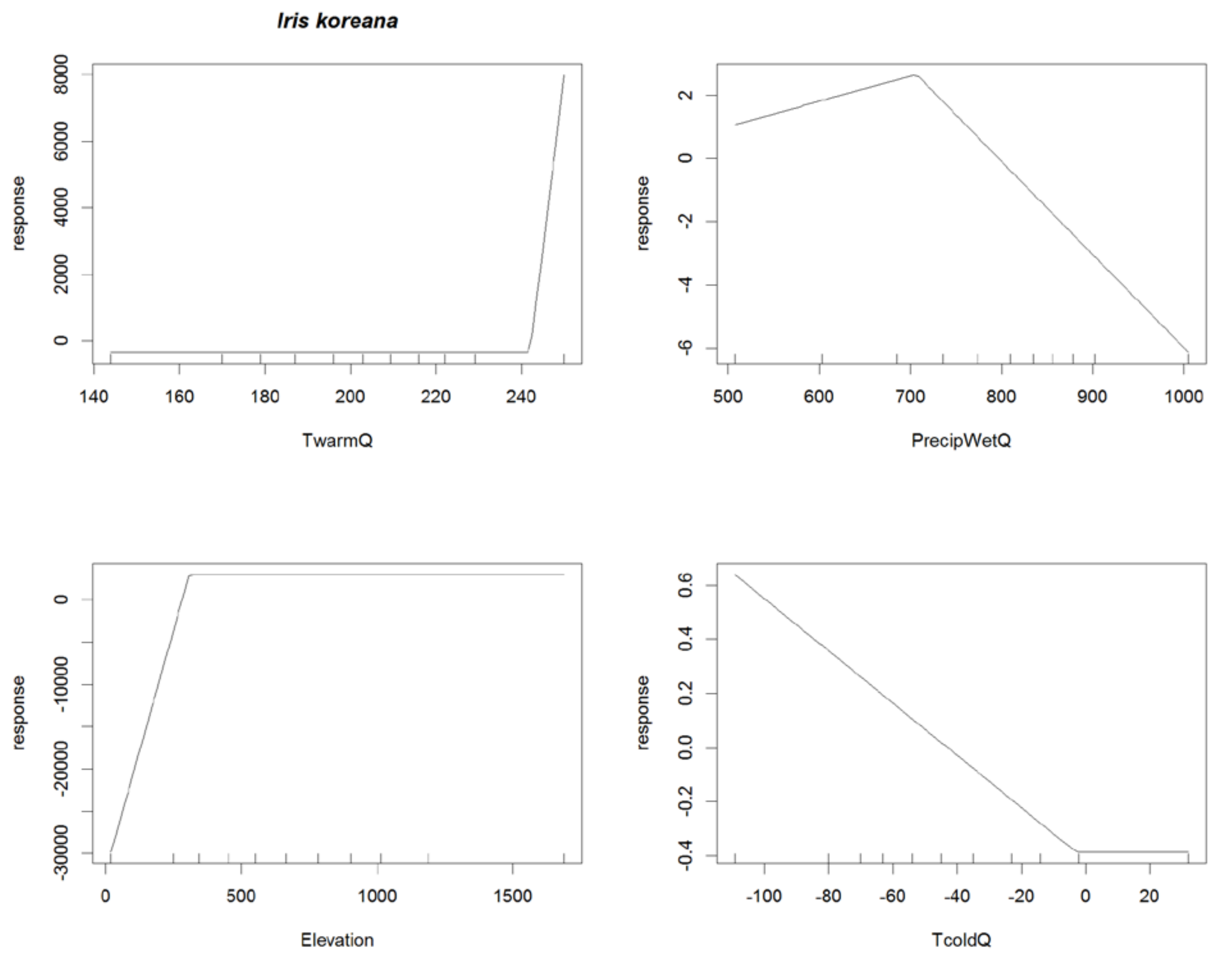

S1 Fig 5. Fitted MARS functions for *Iris koreana* (Yellow Iris): Endangered Species Class Ⅱ (2 occurrence records). (TwarmQ: Mean temperature of warmest quarter, PrecipWetQ: Precipitation of wettest quarter, TcoldQ: Mean temperature of coldest quarter)
