# Supplementary material for: Mapping National Plant Biodiversity Patterns in South Korea with the MARS Species Distribution Model
Source: PLoS One. 2016 Mar 1;11(3):e0149511. doi: 10.1371/journal.pone.0149511 (PMC4773094; doi:10.1371/journal.pone.0149511)
Supplement: S2 Fig — (PDF) [file pone.0149511.s003.pdf]

S2 Fig. The Baekdudaegan Mountain Range and the national parks on the species richness maps of South Korea.

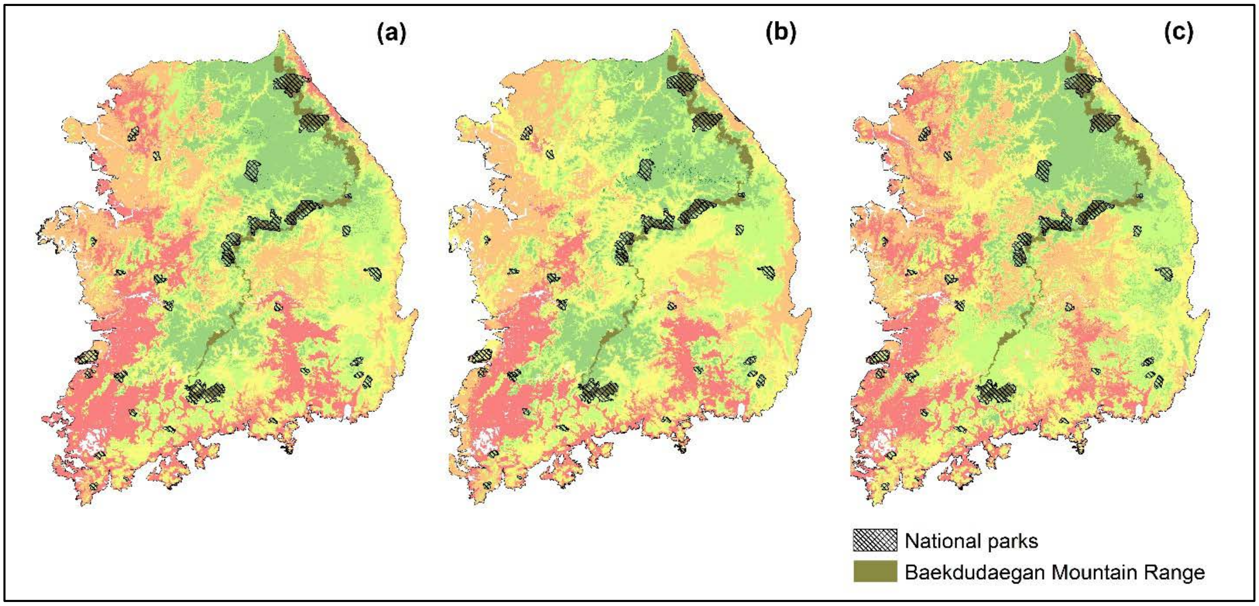

S2 Fig. Overlaid maps of Baekdudaegan Mountain Range (an area of known high plant diversity) and all national parks with species richness maps: (a) All species, (b) Endangered and endemic species, (c) Range-size rarity weighted maps from all species (details and legends in the text). Non-zero values are grouped into five classes (from red (lowest) to green (highest)), each class contains equal numbers of grid cells (quintile).

The table below illustrates the proportion of the Baekdudaegan Mountain Range and the national parks in each class of the species richness maps of South Korea.

All species: quintile classification of South Korea (each 20%)	% of the Baekdudaegan Mountain Range	% of the national parks	Endangered and endemic species: quintile classification of South Korea (each 20%)	% of the Baekdudaegan Mountain Range	% of the national parks
7 – 28	0.07%	2.54%	1 – 4	0%	1.23%
29 – 36	2.39%	6.55%	5 – 6	0.06%	3.38%
37 – 45	9.50%	16.61%	7 – 9	8.32%	17.72%
46 – 65	20.76%	29.92%	10 – 15	35.22%	37.77%
66 - 107	67.29%	44.38%	16 - 26	56.40%	39.89%
