# Supplementary material for: Mapping National Plant Biodiversity Patterns in South Korea with the MARS Species Distribution Model
Source: PLoS One. 2016 Mar 1;11(3):e0149511. doi: 10.1371/journal.pone.0149511 (PMC4773094; doi:10.1371/journal.pone.0149511)
Supplement: S3 Fig — Species richness maps by number of occurrence points from each group category, using the one standard deviation thresholds: (a) All species, (b) Endangered and endemic species, (c) Range-size rarity weighted maps from all species. (PDF) [file pone.0149511.s004.pdf]

**S3 Fig. Species richness maps by number of occurrence points from each group category, using the one standard deviation thresholds: (a) All species, (b) Endangered and endemic species, (c) Range-size rarity weighted maps from all species.** These maps can be compared with Figure 3 in the text.

To consider the contributions of species with few observations to the national species richness patterns, we divided modeled species based on the number of occurrence points. We took the modeled ranges for 106 species (53% out of 199 species) with less than or equal to 5 occurrence records (S3 Fig. 1) and others (93 species with 6 or more points) (S3 Fig. 2) and took the difference (S3 Fig. 3).

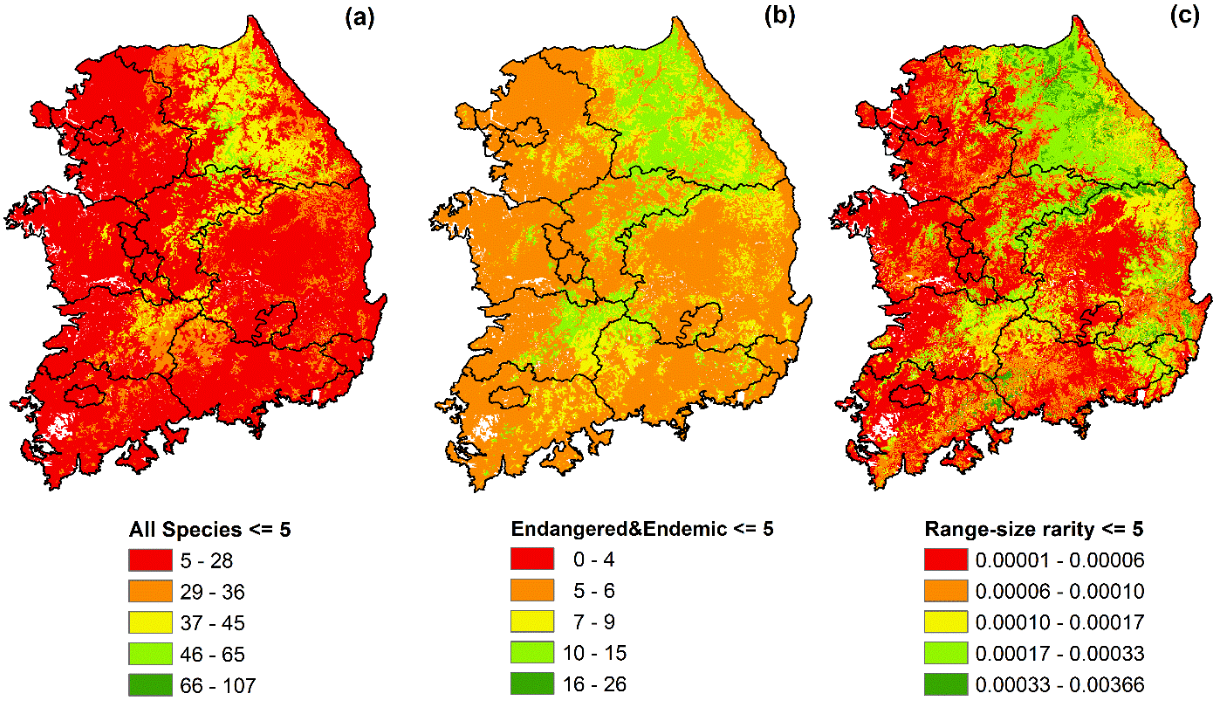


S3 Fig 1. Species richness maps with species less than or equal to 5 occurrence points.

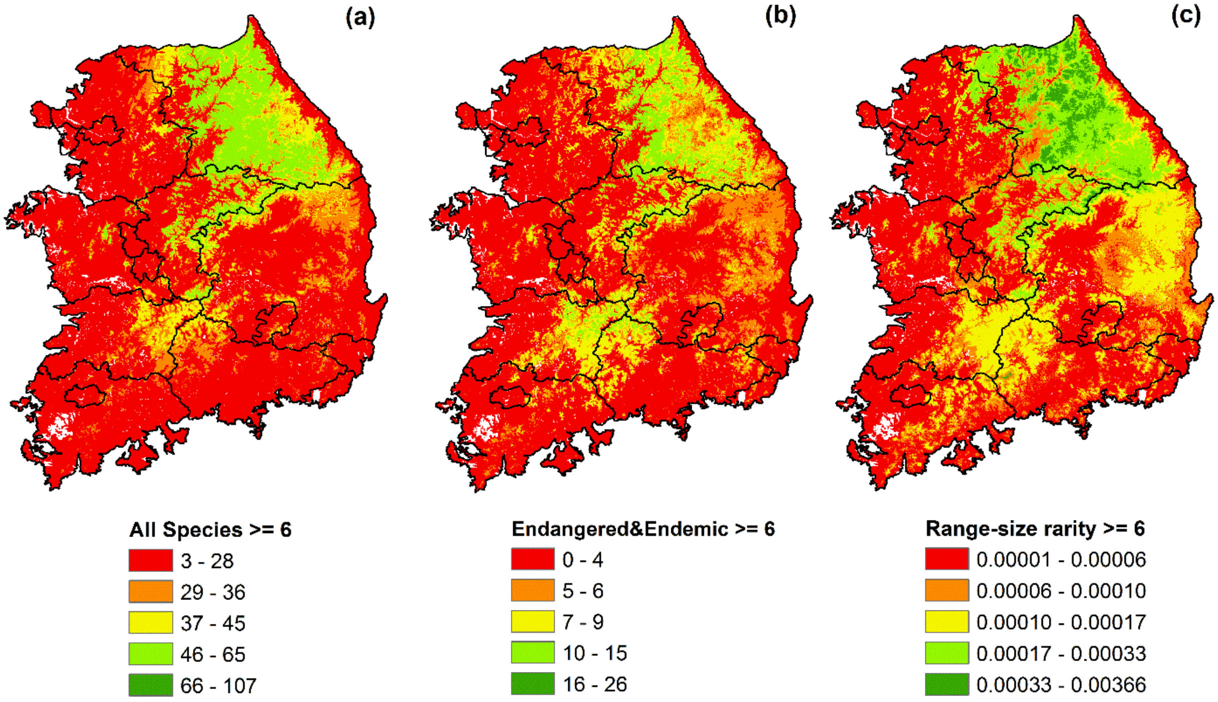


S3 Fig 2. Species richness maps with species more than 5 occurrence points.

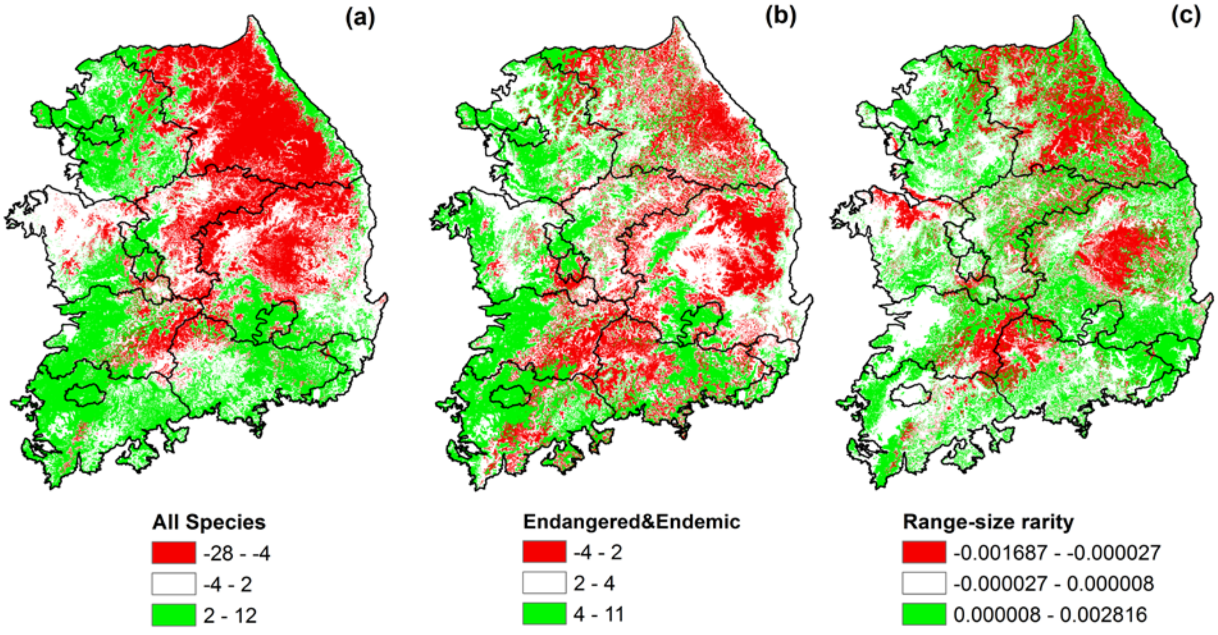


S3 Fig. 3. The difference between species richness maps with 5 or fewer occurrence points (S3 Fig. 1) and species richness maps of species with 6 or more occurrence points (S3 Fig. 2). Higher species richness in green shows where the species with 5 or fewer records produce greater

species richness, and the red shows where the more common (6 or more records) species richness map contributes more than the rare species. Several locations along the western shore and the southeast portion of the country show greater species richness when the species with the fewest records are included.

As S3 Fig. 3 shows, the inclusion of species with few records provides distinct patterns of species richness for our overall analyses that amplify and extend the national species richness patterns from those using species with higher numbers of records.
