# Supplementary material for: Mapping National Plant Biodiversity Patterns in South Korea with the MARS Species Distribution Model
Source: PLoS One. 2016 Mar 1;11(3):e0149511. doi: 10.1371/journal.pone.0149511 (PMC4773094; doi:10.1371/journal.pone.0149511)
Supplement: S1 Table — (PDF) [file pone.0149511.s005.pdf]

S1 Table. The list of modeled plant species.

Modeled species	# of occurrence points used in modeling	# of points in the independent dataset	# of included points: Mean	# of included points: Median	# of included points: 1SD	# of included points: Max SSS	Endangered species	Endemic species
Abelia coreana	7	27	14	14	5	17		
Abies koreana	3	2	0	0	0	0		V
Acanthopanax chiisanensis	18	14	7	8	8	11		V
Acer barbinerve	11	23	4	4	13	12		
Acer mono	3	3	2	2	0	2		
Acer tegmentosum	19	21	13	12	17	19		
Acer ukurunduense	17	20	3	2	13	11		
Aconitum chiisanense	2	0	0	0	0	0		
Aconitum koreanum	7	12	9	8	8	11	V	
Aconitum trilobum	10	9	1	1	2	6	V	V
Adenophora grandiflora	2	5	0	0	2	0		
Aegopodium alpestre	2	5	0	0	1	1		
Ajuga spectabilis	7	22	8	8	9	15		V
Allium senescens	4	2	0	0	0	0		
Allium victorialis var. *platyphyllum*	2	0	0	0	0	0		
Anemone koraiensis	20	21	3	3	10	7		V
Anemone narcissiflora	3	0	0	0	0	0		
Anemone reflexa	31	0	0	0	0	0		
Angelica gigas	15	2	0	0	0	0		
Angelica tenuissima	7	13	4	1	6	7		
Arisaema heterophyllum	3	12	1	1	4	5		
Aristolochia contorta	21	73	19	28	50	51		
Aristolochia manshuriensis	28	39	9	15	29	19		
Asarum maculatum	2	15	6	6	1	8		V
Astragalus membranaceus	4	8	1	1	2	2		
Berchemia berchemiaefolia	29	3	0	0	2	2		
Bistorta alopecuroides	2	0	0	0	0	0		

Bupleurum euphorbioides	5	0	0	0	0	0		V
Bupleurum falcatum	7	53	34	36	34	34		
Buxus microphylla var. *koreana*	11	69	10	12	17	19		
Calanthe discolor	2	0	0	0	0	0		
Campylotropis macrocarpa	6	9	1	1	1	1		
Cardamine koreana	4	0	0	0	0	0		V
Cardamine lyrate	2	19	0	0	0	0		
Carex kujujana	3	0	0	0	0	0		
Carex ligulata var. *austrokoreensis*	2	0	0	0	0	0		
Carpinus coreana	2	63	6	6	7	4		
Celtis choseniana	12	4	1	1	1	2		V
Cephalotaxus koreana	3	50	11	17	48	17		
Chionanthus retusa	2	18	2	2	0	2		
Chrysosplenium ramosum	6	10	0	1	8	1		
Cimicifuga heracleifolia	65	10	2	2	1	2		
Cirsium chanroenicum	8	23	12	10	14	13		
Cirsium setidens	5	130	44	54	70	73		
Cirsium vlassovianum	4	3	1	1	0	1		
Clematis brachyura	3	60	4	4	9	6		V
Clematis chiisanensis	5	0	0	0	0	0		
Clematis patens	6	2	0	0	0	0		
Clintonia udensis	4	2	1	1	1	1		
Cnidium tachiroei	5	0	0	0	0	0		
Codonopsis lanceolata	49	128	10	11	103	30		
Convallaria keiskei	9	245	52	35	73	105		
Corydalis grandicalyx	2	7	0	0	7	0		V
Corydalis maculata	5	2	0	0	0	0		
Corylopsis coreana	23	30	19	19	21	27		
Crataegus komarovii	2	0	0	0	0	0		
Cremastra appendiculata	3	4	2	2	4	4		

Crypsinus hastatus	8	18	3	4	18	5		
Cymbidium goeringii	3	85	8	10	18	14		
Cymbidium nipponicum	2	0	0	0	0	0	V	
Cypripedium macranthum	11	5	5	5	5	5		
Daphne kamtschatica	3	2	0	0	0	0		
Delphinium maackianum	2	4	3	3	0	3		
Deutzia coreana	4	229	32	31	16	49		
Deutzia paniculata	2	5	0	0	5	0		V
Dianthus superbus var. *longicalycinus*	5	63	3	7	12	10		
Dicentra spectabilis	39	67	5	6	17	34		
Dipsacus japonicus	5	14	0	0	2	0		
Disporum ovale	18	0	0	0	0	0		
Disporum sessile	4	161	28	30	33	59		
Dryopteris crassirhizoma	5	140	14	22	40	48		
Echinosophora koreensis	2	7	0	0	7	0		V
Epimedium koreanum	13	17	4	5	17	15		
Equisetum hyemale	12	30	15	15	19	23		
Eranthis stellata	11	49	1	0	29	1		
Euonymus pauciflorus	17	36	15	20	22	14		
Eurya japonica	6	91	65	70	41	83		
Filipendula formosa	3	0	0	0	0	0		V
Filipendula glaberrima	19	76	20	31	34	40		
Forsythia ovata	4	0	0	0	0	0		V
Forsythia saxatilis	3	4	0	0	0	0		V
Galium boreale var. *vulgare*	4	9	0	0	2	3		
Gastrodia elata	37	17	3	5	10	12		
Halenia corniculata	3	0	0	0	0	0	V	
Hanabusaya asiatica	20	15	7	7	9	11		V
Hemerocallis middendorfil	5	5	0	0	0	0		
Hovenia dulcis	10	17	3	3	14	14		

Hylomecon hylomeconoides	35	22	11	10	20	21		V
Ilex cornuta	2	8	1	1	8	3		
Ilex macropoda	29	77	41	43	38	49		
Iris ensata var. *spontanea*	3	21	0	1	21	4		
Iris koreana	2	2	1	1	2	2	V	V
Iris odaesanensis	24	14	2	4	10	6		V
Iris ruthenica	8	11	4	4	3	5	V	
Iris savatieri	4	41	9	10	14	18		
Isopyrum mandshuricum	2	6	1	1	4	3		
Isopyrum raddeanum	3	11	3	2	9	9		
Jeffersonia dubia	4	6	1	1	4	5		
Kalopanax pictus	69	164	19	22	69	25		
Koelreuteria paniculata	7	25	1	0	2	8		
Larix gmelini var. *principisruprechtii*	4	13	4	4	2	6		
Leontice microrhyncha	6	0	0	0	0	0		
Leontopodium coreanum	2	0	0	0	0	0		V
Leontopodium japonicum	5	3	1	1	0	1		
Lilium callosum	2	7	0	0	1	0		
Lilium cernum	20	19	9	10	12	14		
Lilium distichum	30	93	58	64	71	81		
Lonicera chrysaniha	7	8	0	0	4	1		
Lonicera harai	3	33	1	1	3	4		
Lonicera sachalinensis	2	2	0	0	0	0		
Lonicera subhispida	3	10	1	1	0	1		
Lonicera subsessilis	14	65	17	17	40	21		V
Lonicera vesicaria	3	5	1	1	0	1		
Loranthus tanakae	2	0	0	0	0	0		
Lysimachia coreana	9	17	1	1	3	1		V
Machilus thunbergii	3	19	2	2	2	5		
Magnolia kobus	2	3	0	0	0	0		
Megaleranthis saniculifolia	6	0	0	0	0	0		V
Melampyrum setaceum var. nakaianum	6	27	6	7	6	11		

Moehringia lateriflora	6	4	1	1	1	2		
Monotropa hypopithys	3	4	1	1	1	1		
Monotropa uniflora	4	10	1	1	2	2		
Nymphoides peltata	20	5	0	0	0	0		
Oplopanax elatus	16	0	0	0	0	0		
Paeonia japonica	5	17	8	9	13	9		
Paeonia obovata	17	3	0	1	1	1	V	
Patrina saniculaefolia	26	41	12	12	22	13		
Patrinia rupestris	7	22	3	3	17	7		
Paulownia coreana	12	151	48	60	113	130		V
Phellodendron amurense	25	30	1	1	19	21		
Pimpinella brachycarpa	2	178	14	14	54	178		
Pinellia tripartia	2	3	3	3	3	3		
Pinus pumila	2	0	0	0	0	0		
Pleuropterus cilinervis	2	14	0	0	1	14		
Poa viridula	2	17	0	0	5	1		
Polygonatum stenophyllum	3	3	1	1	0	1	V	
Populus maximowiczii	4	2	0	0	0	0		
Prunus davidiana	13	26	12	12	13	14		
Prunus maackii	3	12	2	2	3	2		
Prunus yedoensis	32	46	5	2	9	21		
Pulsatilla koreana	8	219	33	37	56	72		
Quercus serrata	2	657	20	20	65	53		
Ranunculus kazusensis	2	0	0	0	0	0	V	
Rhamnus parvifolia	3	11	3	3	4	4		
Rhododendron brachycarpum	8	0	0	0	0	0		
Rhododendron micranthum	22	22	14	15	11	20		
Rhododendron tschonoskii	12	3	0	0	3	2		
Rodgersia podophylla	59	62	24	24	36	41		
Rosa davurica	2	8	0	0	0	0		
Rosa marretii	3	0	0	0	0	0		
Salvia chanroenica	5	36	7	5	9	11		V

Sanguisorba hakusanensis	4	11	0	0	5	5		V
Sanguisorba longifolia	6	3	0	0	3	1		
Sapium japonicum	4	103	7	6	12	18		
Saussurea calcicola	3	7	0	2	7	5		V
Saussurea eriophylla	2	7	2	2	6	2		V
Saxifraga punctata	15	4	0	3	3	3		
Scabiosa mansenensis	7	0	0	0	0	0		
Schizopepon bryoniaefolius	10	26	2	2	8	10		
Scopolia japonica	25	42	3	3	26	19		
Scrophularia koraiensis	4	29	5	5	4	5		
Sedum rotundifolium	8	0	0	0	0	0		
Sedum zokuriense	2	0	0	0	0	0		V
Silene koreana	6	7	1	1	5	4		
Smilacina bicolor	16	0	0	0	0	0		V
Sorbus amurensis	8	7	0	1	6	1		
Sorbus commixta	14	24	3	3	11	5		
Spiraea miyabei	3	9	0	0	2	0		
Spiraea salicifolia	18	114	75	65	76	71		
Stewartia koreana	23	44	22	27	23	33		V
Symplocarpus nipponicus	17	2	0	0	0	0		
Symplocarpus renifolius	13	14	6	6	12	6		
Syneilesis palmata	2	344	9	9	5	10		
Syringa velutina var. *kamibayashii*	2	0	0	0	0	0		
Syringa wolfi	31	23	9	9	13	9		
Taxus cuspidata	12	33	3	4	33	4		
Thalictrum coreanum	4	2	0	0	1	1	V	
Thalictrum rochebrunianum	2	19	1	1	0	1		V
Thuja koraiensis	9	0	0	0	0	0		
Thuja orientalis	8	46	13	16	20	25		
Thymus quinquecostatus	4	8	1	1	4	5		
Tilia taquetii	3	11	0	0	0	0		
Torreya nucifera	4	20	6	12	15	13		
Tricyrtis dilatata	18	54	7	4	22	27		

Trientalis europaea	2	2	1	1	1	1	V	
Trillium kamtschaticum	21	15	2	6	15	13		
Trillium tschonoskii	6	2	0	0	1	1		
Ulmus macrocarpa	4	18	0	0	2	3		
Vaccinium koreanum	6	112	19	18	54	20		V
Vicia bungei	2	7	1	1	7	1		
Viola albida	2	41	0	0	0	0		
Viola diamantica	37	51	22	16	26	27		
Viola variegata	8	245	61	40	91	85		
Waldsteinia ternata	5	9	1	1	4	5		
Weigela florida	8	168	43	41	76	46		
Weigela subsessilis	3	500	29	24	35	53		V
Wistaria floribunda	4	44	5	7	43	11		
Youngia chelidoniifoli	4	89	5	5	15	17		
Zanthoxylum piperitum	3	240	38	32	25	47		
