# Supplementary material for: Mapping National Plant Biodiversity Patterns in South Korea with the MARS Species Distribution Model
Source: PLoS One. 2016 Mar 1;11(3):e0149511. doi: 10.1371/journal.pone.0149511 (PMC4773094; doi:10.1371/journal.pone.0149511)
Supplement: S2 Table — (PDF) [file pone.0149511.s006.pdf]

S2 Table. Elevation variations on each species occurrence points between 30 m and 1 km elevation rasters, and distance between points for each species.

S2 Table 1. The variation in elevation of each species' occurrence points between 30 m and 1 km elevation rasters.

species	*30m Minimum Elevation (m)*	*1km Minimum Elevation (m)*	*30m Maximum Elevation (m)*	*1km Maximum Elevation (m)*	*30m Elevation Range (m)*	*1km Elevation Range (m)*	*30m Mean Elevation (m)*	*1km Mean Elevation (m)*
Abelia coreana	286	302	1388	1290	1102	988	780	746
Abies koreana	1324	1139	1338	1229	14	90	1329	1169
Acanthopanax chiisanensis	382	340	1193	1081	811	741	730	708
Acer barbinerve	614	690	1403	1427	789	737	974	970
Acer mono	226	222	337	503	111	281	287	397
Acer tegmentosum	374	478	1372	1218	998	739	885	894
Acer ukurunduense	704	775	1295	1223	591	448	1031	1038
Aconitum chiisanense	429	551	738	615	309	64	584	583
Aconitum koreanum	340	327	955	794	615	467	529	525
Aconitum trilobum	381	355	665	689	284	333	498	533
Adenophora grandiflora	1125	1151	1410	1288	285	137	1268	1220
Aegopodium alpestre	841	754	1310	1237	469	483	1076	995
Ajuga spectabilis	415	339	933	869	518	530	653	570
Allium senescens	277	354	1250	1134	973	780	659	573
Allium victorialis var. platyphyllum	1098	1167	1354	1229	256	62	1226	1198
Anemone koraiensis	393	442	1487	1427	1094	986	938	939
Anemone narcissiflora	767	801	1321	1210	554	409	1063	1011
Anemone reflexa	301	371	1437	1330	1136	959	785	783
Angelica gigas	361	320	1135	1102	774	782	835	810
Angelica tenuissima	954	776	1178	1082	224	305	1040	934
Arisaema heterophyllum	198	348	697	569	499	221	402	430
Aristolochia contorta	127	179	605	657	478	478	329	376
Aristolochia manshuriensis	226	289	1089	1152	863	864	638	668
Asarum maculatum	274	287	885	724	611	437	580	505
Astragalus membranaceus	289	288	624	746	335	458	433	495
Berchemia berchemiaefolia	163	261	610	612	447	351	351	427
Bistorta alopecuroides	439	316	731	560	292	244	585	438
Bupleurum euphorbioides	593	413	1192	1003	599	591	867	799
Bupleurum falcatum	251	149	963	908	712	759	660	560
Buxus microphylla var. koreana	246	231	671	615	425	385	357	404
Calanthe discolor	149	199	252	222	103	24	201	210
Campylotropis macrocarpa	197	190	461	424	264	235	294	316
Cardamine koreana	354	378	982	930	628	552	645	683
Cardamine lyrate	815	841	865	841	50	0	840	841
Carex kujujana	160	91	273	377	113	286	228	276
Carex ligulata var. austrokoreensis	49	186	309	405	260	219	179	296
Carpinus coreana	358	214	365	252	7	38	362	233
Celtis choseniana	183	172	965	804	782	632	574	543
Cephalotaxus koreana	215	312	487	487	272	175	363	429
Chionanthus retusa	113	115	128	124	15	9	121	120
Chrysosplenium ramosum	349	441	1024	1095	675	653	775	866
Cimicifuga heracleifolia	182	189	1446	1351	1264	1161	742	721
Cirsium chanroenicum	403	434	1084	962	681	527	773	724
Cirsium setidens	233	263	711	631	478	368	433	400
Cirsium vlassovianum	511	537	1032	911	521	374	845	767
Clematis brachyura	310	312	444	358	134	46	355	335

Clematis chiisanensis	*335*	*341*	*1540*	*1371*	*1205*	*1029*	*961*	*927*
Clematis patens	*160*	*177*	*907*	*856*	*747*	*679*	*357*	*363*
Clintonia udensis	*373*	*352*	*1222*	*1179*	*849*	*827*	*976*	*879*
Cnidium tachiroei	*407*	*442*	*1251*	*1179*	*844*	*737*	*773*	*751*
Codonopsis lanceolata	*151*	*132*	*1087*	*1042*	*936*	*910*	*560*	*564*
Convallaria keiskei	*298*	*296*	*896*	*776*	*598*	*480*	*494*	*430*
Corydalis grandicalyx	*1310*	*1237*	*1471*	*1325*	*161*	*88*	*1391*	*1281*
Corydalis maculata	*287*	*392*	*866*	*917*	*579*	*525*	*656*	*708*
Corylopsis coreana	*224*	*273*	*1114*	*1232*	*890*	*959*	*639*	*648*
Crataegus komarovii	*193*	*229*	*450*	*490*	*257*	*261*	*322*	*360*
Cremastra appendiculata	*260*	*279*	*274*	*366*	*14*	*86*	*269*	*319*
Crypsinus hastatus	*102*	*159*	*346*	*441*	*244*	*282*	*200*	*300*
Cymbidium goeringii	*188*	*118*	*296*	*319*	*108*	*201*	*234*	*187*
Cymbidium nipponicum	*221*	*296*	*389*	*319*	*168*	*23*	*305*	*308*
Cypripedium macranthum	*268*	*219*	*1343*	*1229*	*1075*	*1010*	*798*	*756*
Daphne kamtschatica	*731*	*657*	*830*	*913*	*99*	*256*	*790*	*816*
Delphinium maackianum	*858*	*756*	*915*	*778*	*57*	*22*	*887*	*767*
Deutzia coreana	*285*	*403*	*557*	*503*	*272*	*100*	*415*	*455*
Deutzia paniculata	*339*	*375*	*717*	*575*	*378*	*199*	*528*	*475*
Dianthus superbus var. longicalycinus	*358*	*312*	*838*	*816*	*480*	*503*	*601*	*546*
Dicentra spectabilis	*144*	*147*	*876*	*879*	*732*	*732*	*459*	*526*
Dipsacus japonicus	*305*	*484*	*926*	*707*	*621*	*223*	*579*	*554*
Disporum ovale	*314*	*322*	*1347*	*1229*	*1033*	*907*	*969*	*916*
Disporum sessile	*181*	*222*	*698*	*625*	*517*	*402*	*402*	*380*
Dryopteris crassirhizoma	*339*	*337*	*897*	*970*	*558*	*634*	*575*	*552*
Echinosophora koreensis	*338*	*383*	*533*	*418*	*195*	*34*	*436*	*401*
Epimedium koreanum	*40*	*44*	*806*	*629*	*766*	*586*	*357*	*324*
Equisetum hyemale	*579*	*653*	*1104*	*1081*	*525*	*428*	*859*	*927*
Eranthis stellata	*267*	*416*	*1295*	*1107*	*1028*	*691*	*757*	*784*
Euonymus pauciflorus	*214*	*152*	*1326*	*1178*	*1112*	*1026*	*674*	*618*
Eurya japonica	*162*	*77*	*424*	*408*	*262*	*331*	*249*	*228*
Filipendula formosa	*1053*	*933*	*1620*	*1512*	*567*	*580*	*1412*	*1284*
Filipendula glaberrima	*221*	*269*	*1532*	*1427*	*1311*	*1159*	*868*	*844*
Forsythia ovata	*441*	*356*	*1038*	*812*	*597*	*456*	*825*	*693*
Forsythia saxatilis	*127*	*254*	*404*	*452*	*277*	*198*	*288*	*328*
Galium boreale var. vulgare	*213*	*180*	*575*	*438*	*362*	*258*	*333*	*322*
Gastrodia elata	*175*	*332*	*1258*	*1165*	*1083*	*833*	*591*	*569*
Halenia corniculata	*978*	*988*	*1202*	*1143*	*224*	*155*	*1076*	*1047*
Hanabusaya asiatica	*449*	*488*	*1320*	*1270*	*871*	*782*	*973*	*919*
Hemerocallis middendorfil	*906*	*796*	*1262*	*1084*	*356*	*288*	*1087*	*950*
Hovenia dulcis	*225*	*236*	*891*	*903*	*666*	*666*	*467*	*499*
Hylomecon hylomeconoides	*184*	*185*	*1322*	*1214*	*1138*	*1029*	*399*	*468*
Ilex cornuta	*20*	*54*	*124*	*77*	*104*	*23*	*72*	*65*
Ilex macropoda	*116*	*172*	*920*	*845*	*804*	*672*	*606*	*589*
Iris ensata var. spontanea	*285*	*287*	*955*	*741*	*670*	*454*	*592*	*525*
Iris koreana	*615*	*565*	*931*	*926*	*316*	*361*	*773*	*745*
Iris odaesanensis	*246*	*359*	*1554*	*1427*	*1308*	*1068*	*871*	*842*
Iris ruthenica	*96*	*90*	*814*	*862*	*718*	*772*	*477*	*525*
Iris savatieri	*181*	*207*	*827*	*759*	*646*	*552*	*544*	*486*
Isopyrum mandshuricum	*287*	*389*	*521*	*597*	*234*	*209*	*404*	*493*
Isopyrum raddeanum	*820*	*891*	*1104*	*1013*	*284*	*121*	*948*	*951*
Jeffersonia dubia	*162*	*201*	*504*	*438*	*342*	*237*	*346*	*359*
Kalopanax pictus	*141*	*54*	*1242*	*1082*	*1101*	*1028*	*650*	*660*
Koelreuteria paniculata	*33*	*26*	*903*	*965*	*870*	*939*	*218*	*210*
Larix gmelini var principisruprechtii	*596*	*466*	*737*	*719*	*141*	*253*	*667*	*592*
Leontice microrhyncha	*981*	*1057*	*1197*	*1178*	*216*	*121*	*1092*	*1097*
Leontopodium coreanum	*1109*	*998*	*1272*	*1135*	*163*	*138*	*1191*	*1067*
Leontopodium japonicum	*961*	*791*	*1221*	*1087*	*260*	*296*	*1125*	*990*
Lilium callosum	*320*	*355*	*386*	*465*	*66*	*110*	*353*	*410*

Lilium cernum	529	465	1310	1189	781	724	892	779
Lilium distichum	150	177	1280	1184	1130	1007	766	730
Lonicera chrysaniha	365	355	1543	1419	1178	1064	873	868
Lonicera harai	466	642	802	716	336	74	634	668
Lonicera sachalinensis	468	558	1274	1210	806	652	871	884
Lonicera subhispida	425	452	564	579	139	127	497	517
Lonicera subsessilis	263	289	1205	1154	942	865	700	679
Lonicera vesicaria	998	969	1352	1203	354	234	1189	1058
Loranthus tanakae	740	713	978	878	238	165	859	795
Lysimachia coreana	224	283	1164	1143	940	860	641	646
Machilus thunbergii	64	125	262	384	198	259	149	236
Magnolia kobus	77	115	198	216	121	101	138	166
Megaleranthis saniculifolia	363	365	991	1007	628	642	793	823
Melampyrum setaceum var. nakaianum	469	423	989	936	520	513	767	753
Moehringia lateriflora	333	333	761	757	428	424	524	580
Monotropa hypopithys	220	209	602	606	382	397	474	402
Monotropa uniflora	619	545	1250	1184	631	640	1011	928
Nymphoides peltata	31	34	102	149	71	115	45	51
Oplopanax elatus	647	679	1666	1539	1019	860	1165	1147
Paeonia japonica	267	257	1013	921	746	665	677	637
Paeonia obovata	292	396	1270	1254	978	857	878	841
Patrina saniculaefolia	170	132	1312	1229	1142	1098	896	822
Patrinia rupestris	487	413	1076	1031	589	618	719	686
Paulownia coreana	123	109	539	609	416	500	376	398
Phellodendron amurense	271	287	1048	1107	777	819	625	669
Pimpinella brachycarpa	761	648	921	1030	160	383	841	839
Pinellia tripartia	237	203	456	408	219	205	347	305
Pinus pumila	1174	921	1669	1481	495	561	1422	1201
Pleuropterus cilinervis	212	222	510	366	298	144	361	294
Poa viridula	307	231	379	378	72	147	343	305
Polygonatum stenophyllum	243	302	333	344	90	42	278	329
Populus maximowiczii	279	343	657	710	378	367	452	530
Prunus davidiana	140	193	754	664	614	471	422	425
Prunus maackii	463	511	1017	1107	554	596	767	835
Prunus yedoensis	97	105	404	452	307	347	163	195
Pulsatilla koreana	183	160	470	464	287	305	324	346
Quercus serrata	356	289	441	337	85	48	399	313
Ranunculus kazusensis	8	5	26	19	18	14	17	12
Rhamnus parvifolia	663	666	928	794	265	128	784	714
Rhododendron brachycarpum	1042	992	1343	1352	301	360	1224	1149
Rhododendron micranthum	229	198	1146	952	917	754	709	642
Rhododendron tschonoskii	953	799	1679	1539	726	740	1325	1212
Rodgersia podophylla	305	412	1383	1315	1078	903	821	816
Rosa davurica	337	378	1332	1246	995	868	835	812
Rosa marretii	716	775	1513	1427	797	652	1187	1148
Salvia chanroenica	335	352	744	683	409	332	538	516
Sanguisorba hakusanensis	966	776	1234	1232	268	455	1164	1062
Sanguisorba longifolia	306	145	1668	1539	1362	1394	816	735
Sapium japonicum	222	203	326	304	104	101	297	247
Saussurea calcicola	683	644	1366	1215	683	571	994	922
Saussurea eriophylla	324	338	517	501	193	163	421	420
Saxifraga punctata	500	380	1227	1228	727	848	762	826
Scabiosa mansenensis	190	192	804	908	614	716	548	551
Schizopepon bryoniaefolius	508	551	1092	1057	584	506	834	892
Scopolia japonica	169	287	1006	942	837	655	579	606
Scrophularia koraiensis	1057	991	1315	1180	258	189	1199	1104
Sedum rotundifolium	106	184	547	694	441	510	374	445
Sedum zokuriense	523	423	991	823	468	400	757	623
Silene koreana	226	243	730	638	504	395	391	408

Smilacina bicolor	*1230*	*1237*	*1688*	*1512*	*458*	*276*	*1491*	*1382*
Sorbus amurensis	*367*	*451*	*1406*	*1351*	*1039*	*899*	*958*	*939*
Sorbus commixta	*305*	*402*	*1474*	*1310*	*1169*	*908*	*954*	*897*
Spiraea miyabei	*460*	*530*	*1003*	*988*	*543*	*458*	*689*	*778*
Spiraea salicifolia	*200*	*209*	*1158*	*1081*	*958*	*872*	*594*	*618*
Stewartia koreana	*192*	*258*	*1069*	*969*	*877*	*711*	*648*	*619*
Symplocarpus nipponicus	*269*	*286*	*1518*	*1354*	*1249*	*1068*	*886*	*899*
Symplocarpus renifolius	*215*	*236*	*1360*	*1289*	*1145*	*1053*	*764*	*760*
Syneilesis palmata	*349*	*332*	*371*	*358*	*22*	*25*	*360*	*345*
Syringa velutina var. kamibayashii	*594*	*387*	*1079*	*991*	*485*	*605*	*837*	*689*
Syringa wolfi	*217*	*310*	*1558*	*1427*	*1341*	*1116*	*1063*	*1023*
Taxus cuspidata	*440*	*338*	*1491*	*1427*	*1051*	*1089*	*1186*	*1086*
Thalictrum coreanum	*307*	*385*	*857*	*650*	*550*	*265*	*497*	*504*
Thalictrum rochebrunianum	*490*	*334*	*583*	*409*	*93*	*76*	*537*	*372*
Thuja koraiensis	*977*	*801*	*1676*	*1396*	*699*	*595*	*1175*	*1089*
Thuja orientalis	*187*	*223*	*1491*	*1343*	*1304*	*1120*	*505*	*449*
Thymus quinquecostatus	*384*	*557*	*964*	*730*	*580*	*173*	*642*	*622*
Tilia taquetii	*211*	*202*	*390*	*366*	*179*	*164*	*271*	*264*
Torreya nucifera	*95*	*153*	*351*	*382*	*256*	*228*	*224*	*284*
Tricyrtis dilatata	*114*	*109*	*905*	*972*	*791*	*863*	*394*	*400*
Trientalis europaea	*1209*	*1095*	*1232*	*1155*	*23*	*61*	*1221*	*1125*
Trillium kamtschaticum	*764*	*873*	*1577*	*1410*	*813*	*537*	*1172*	*1150*
Trillium tschonoskii	*824*	*892*	*1489*	*1386*	*665*	*494*	*1144*	*1126*
Ulmus macrocarpa	*295*	*396*	*729*	*862*	*434*	*466*	*602*	*641*
Vaccinium koreanum	*600*	*541*	*1167*	*1151*	*567*	*610*	*862*	*792*
Vicia bungei	*133*	*143*	*135*	*218*	*2*	*75*	*134*	*181*
Viola albida	*288*	*289*	*627*	*602*	*339*	*313*	*458*	*446*
Viola diamantica	*340*	*339*	*1177*	*1143*	*837*	*805*	*819*	*805*
Viola variegata	*216*	*213*	*573*	*467*	*357*	*254*	*379*	*354*
Waldsteinia ternata	*214*	*228*	*1349*	*1330*	*1135*	*1102*	*816*	*836*
Weigela florida	*178*	*241*	*1134*	*975*	*956*	*734*	*559*	*479*
Weigela subsessilis	*192*	*222*	*351*	*346*	*159*	*124*	*292*	*286*
Wistaria floribunda	*52*	*101*	*710*	*527*	*658*	*426*	*323*	*306*
Youngia chelidoniifoli	*238*	*295*	*1075*	*897*	*837*	*602*	*574*	*549*
Zanthoxylum piperitum	*179*	*153*	*269*	*199*	*90*	*45*	*228*	*177*
Mean	***394***	***397***	***950***	***890***	***556***	***493***	***663***	***642***

S2 Table 2. Distance between occurrence points for each species.

The third column is the number of original resolution (1 km) climate-based raster cells where each species' occurrence points are located. The fourth column is a count of number of climate grid cells that were sampled by more than one occurrence point, which is a signal of possible bias introduced into a species model due to pseudoreplication. When considering species on an individual basis, we recommend discounting the eight species with fewer than five occurrence points that also have one climate grid which was sampled twice.

species	***# of occurrence points***	***# of raster cells the points are found in***	***# possible replicates***	***Minimum Distance (km)***	***Maximum Distance (km)***	***Mean Distance (km)***
Mean				***25.40***	***174.08***	***92.62***
Cardamine lyrate	*2*	*1*	*1*	*0.24*	*0.24*	*0.24*
Thalictrum rochebrunianum	*2*	*2*	*0*	*0.27*	*0.27*	*0.27*
Leontopodium coreanum	*2*	*2*	*0*	*5.73*	*5.73*	*5.73*
Lilium callosum	*2*	*2*	*0*	*6.22*	*6.22*	*6.22*
Deutzia paniculata	*2*	*2*	*0*	*8.06*	*8.06*	*8.06*
Iris koreana	*2*	*2*	*0*	*8.79*	*8.79*	*8.79*
Pimpinella brachycarpa	*2*	*2*	*0*	*9.32*	*9.32*	*9.32*
Calanthe discolor	*2*	*2*	*0*	*9.58*	*9.58*	*9.58*
Sedum zokuriense	*2*	*2*	*0*	*9.65*	*9.65*	*9.65*
Chionanthus retusa	*2*	*2*	*0*	*10.02*	*10.02*	*10.02*
Quercus serrata	*2*	*2*	*0*	*11.72*	*11.72*	*11.72*
Pleuropterus cilinervis	*2*	*2*	*0*	*11.79*	*11.79*	*11.79*
Pinus pumila	*2*	*2*	*0*	*13.78*	*13.78*	*13.78*
Saussurea eriophylla	*2*	*2*	*0*	*13.89*	*13.89*	*13.89*
Loranthus tanakae	*2*	*2*	*0*	*13.99*	*13.99*	*13.99*
Syneilesis palmata	*2*	*2*	*0*	*14.20*	*14.20*	*14.20*
Carpinus coreana	*2*	*2*	*0*	*16.08*	*16.08*	*16.08*
Bistorta alopecuroides	*2*	*2*	*0*	*19.86*	*19.86*	*19.86*
Echinosophora koreensis	*2*	*2*	*0*	*19.87*	*19.87*	*19.87*
Rosa davurica	*2*	*2*	*0*	*32.34*	*32.34*	*32.34*
Ilex cornuta	*2*	*2*	*0*	*41.51*	*41.51*	*41.51*
Larix gmelini var principisruprechtii	*2*	*2*	*0*	*49.18*	*49.18*	*49.18*
Adenophora grandiflora	*2*	*2*	*0*	*51.48*	*51.48*	*51.48*
Aegopodium alpestre	*2*	*2*	*0*	*77.08*	*77.08*	*77.08*
Magnolia kobus	*2*	*2*	*0*	*105.97*	*105.97*	*105.97*
Viola albida	*2*	*2*	*0*	*110.63*	*110.63*	*110.63*
Pinellia tripartia	*2*	*2*	*0*	*118.93*	*118.93*	*118.93*
Carex ligulata var. austrokoreensis	*2*	*2*	*0*	*128.11*	*128.11*	*128.11*
Corydalis grandicalyx	*2*	*2*	*0*	*128.21*	*128.21*	*128.21*
Trientalis europaea	*2*	*2*	*0*	*141.03*	*141.03*	*141.03*
Crataegus komarovii	*2*	*2*	*0*	*145.58*	*145.58*	*145.58*
Vicia bungei	*2*	*2*	*0*	*148.79*	*148.79*	*148.79*
Isopyrum mandshuricum	*2*	*2*	*0*	*150.38*	*150.38*	*150.38*
Aconitum chiisanense	*2*	*2*	*0*	*186.20*	*186.20*	*186.20*
Delphinium maackianum	*2*	*2*	*0*	*210.55*	*210.55*	*210.55*
Lonicera sachalinensis	*2*	*2*	*0*	*213.21*	*213.21*	*213.21*
Ranunculus kazusensis	*2*	*2*	*0*	*216.09*	*216.09*	*216.09*
Poa viridula	*2*	*2*	*0*	*222.44*	*222.44*	*222.44*
Asarum maculatum	*2*	*2*	*0*	*225.81*	*225.81*	*225.81*
Syringa velutina var. kamibayashii	*2*	*2*	*0*	*249.52*	*249.52*	*249.52*
Allium victorialis var. platyphyllum	*2*	*2*	*0*	*253.65*	*253.65*	*253.65*
Cymbidium nipponicum	*2*	*2*	*0*	*261.67*	*261.67*	*261.67*
Abies koreana	*3*	*2*	*1*	*0.13*	*266.54*	*177.71*
Cephalotaxus koreana	*3*	*2*	*1*	*0.34*	*72.58*	*48.39*

Machilus thunbergii	*3*	*3*	*0*	*0.54*	*1.55*	*1.04*
Arisaema heterophyllum	*3*	*3*	*0*	*1.83*	*47.50*	*31.89*
Anemone narcissiflora	*3*	*3*	*0*	*3.34*	*13.47*	*9.39*
Daphne kamtschatica	*3*	*3*	*0*	*3.55*	*6.18*	*5.30*
Acer mono	*3*	*3*	*0*	*3.93*	*280.67*	*187.72*
Filipendula formosa	*3*	*3*	*0*	*3.98*	*41.89*	*27.94*
Clematis brachyura	*3*	*3*	*0*	*4.32*	*12.49*	*8.55*
Polygonatum stenophyllum	*3*	*3*	*0*	*5.01*	*10.66*	*7.34*
Weigela subsessilis	*3*	*3*	*0*	*5.38*	*11.94*	*8.11*
Zanthoxylum piperitum	*3*	*3*	*0*	*8.55*	*17.27*	*12.17*
Rosa marretii	*3*	*3*	*0*	*10.02*	*37.06*	*26.57*
Tilia taquetii	*3*	*3*	*0*	*11.94*	*19.71*	*17.07*
Iris ensata var. spontanea	*3*	*3*	*0*	*12.53*	*150.60*	*100.79*
Cymbidium goeringii	*3*	*3*	*0*	*15.23*	*21.07*	*18.65*
Spiraea miyabei	*3*	*3*	*0*	*17.05*	*188.20*	*127.70*
Halenia corniculata	*3*	*3*	*0*	*18.31*	*57.39*	*40.87*
Forsythia saxatilis	*3*	*3*	*0*	*18.49*	*136.47*	*91.42*
Isopyrum raddeanum	*3*	*3*	*0*	*23.56*	*195.33*	*134.41*
Carex kujujana	*3*	*3*	*0*	*23.70*	*92.25*	*66.68*
Prunus maackii	*3*	*3*	*0*	*33.14*	*51.15*	*41.90*
Lonicera vesicaria	*3*	*3*	*0*	*43.67*	*143.22*	*107.21*
Saussurea calcicola	*3*	*3*	*0*	*50.79*	*98.15*	*75.71*
Lonicera harai	*3*	*3*	*0*	*59.84*	*243.63*	*176.18*
Rhamnus parvifolia	*3*	*3*	*0*	*61.45*	*79.22*	*69.14*
Cremastra appendiculata	*3*	*3*	*0*	*73.50*	*297.01*	*221.42*
Lonicera subhispida	*3*	*3*	*0*	*108.44*	*228.10*	*185.52*
Monotropa hypopithys	*3*	*3*	*0*	*146.32*	*164.14*	*154.64*
Deutzia coreana	*4*	*3*	*1*	*0.15*	*14.23*	*8.27*
Allium senescens	*4*	*3*	*1*	*0.16*	*99.54*	*54.84*
Thymus quinquecostatus	*4*	*3*	*1*	*0.24*	*121.28*	*91.25*
Forsythia ovata	*4*	*3*	*1*	*0.27*	*73.33*	*37.24*
Youngia chelidoniifoli	*4*	*4*	*0*	*0.36*	*5.80*	*3.74*
Sapium japonicum	*4*	*4*	*0*	*0.57*	*20.46*	*12.83*
Sanguisorba hakusanensis	*4*	*4*	*0*	*2.13*	*161.73*	*88.74*
Monotropa uniflora	*4*	*4*	*0*	*2.34*	*187.81*	*124.44*
Astragalus membranaceus	*4*	*4*	*0*	*3.18*	*208.01*	*130.08*
Populus maximowiczii	*4*	*4*	*0*	*4.33*	*73.34*	*44.25*
Disporum sessile	*4*	*4*	*0*	*4.73*	*217.83*	*143.63*
Cardamine koreana	*4*	*4*	*0*	*5.02*	*53.58*	*29.84*
Cirsium vlassovianum	*4*	*4*	*0*	*6.86*	*42.79*	*24.51*
Scrophularia koraiensis	*4*	*4*	*0*	*9.43*	*140.39*	*73.99*
Torreya nucifera	*4*	*4*	*0*	*10.38*	*111.36*	*68.77*
Galium boreale var. vulgare	*4*	*4*	*0*	*11.53*	*49.22*	*29.70*
Thalictrum coreanum	*4*	*4*	*0*	*13.25*	*159.46*	*115.20*
Ulmus macrocarpa	*4*	*4*	*0*	*15.05*	*188.00*	*123.10*
Clintonia udensis	*4*	*4*	*0*	*22.99*	*169.00*	*105.82*
Iris savatieri	*4*	*4*	*0*	*39.95*	*208.78*	*135.32*
Wistaria floribunda	*4*	*4*	*0*	*53.64*	*276.14*	*194.39*
Jeffersonia dubia	*4*	*4*	*0*	*73.97*	*323.28*	*197.60*
Cirsium setidens	*5*	*5*	*0*	*0.12*	*204.67*	*87.40*
Cnidium tachiroei	*5*	*4*	*1*	*0.66*	*288.89*	*135.26*
Leontopodium japonicum	*5*	*5*	*0*	*0.71*	*173.33*	*103.06*
Paeonia japonica	*5*	*5*	*0*	*1.24*	*289.68*	*179.37*
Bupleurum euphorbioides	*5*	*5*	*0*	*2.72*	*150.38*	*61.59*
Clematis chiisanensis	*5*	*5*	*0*	*2.95*	*48.19*	*22.46*
Dianthus superbus var. longicalycinus	*5*	*5*	*0*	*4.29*	*215.19*	*90.07*
Hemerocallis middendorfil	*5*	*5*	*0*	*4.35*	*194.48*	*117.73*
Waldsteinia ternata	*5*	*5*	*0*	*7.00*	*132.80*	*63.62*
Dryopteris crassirhizoma	*5*	*5*	*0*	*7.45*	*130.38*	*56.72*

Corydalis maculata	5	5	0	10.01	96.78	43.72
Salvia chanroenica	5	5	0	18.52	225.70	129.78
Dipsacus japonicus	5	5	0	24.38	191.16	108.27
Melampyrum setaceum var. nakaianum	6	6	0	0.24	212.56	114.27
Chrysosplenium ramosum	6	5	1	0.25	174.05	87.84
Vaccinium koreanum	6	6	0	0.27	211.62	89.41
Leontice microrhyncha	6	5	1	0.35	111.87	59.73
Megaleranthis saniculifolia	6	6	0	0.80	93.92	58.85
Silene koreana	6	6	0	4.48	189.73	67.39
Campylotropis macrocarpa	6	6	0	4.75	139.89	65.22
Clematis patens	6	6	0	5.58	204.60	119.85
Moehringia lateriflora	6	6	0	7.93	246.83	95.49
Eurya japonica	6	6	0	10.09	214.78	111.66
Trillium tschonoskii	6	6	0	13.90	125.99	61.22
Sanguisorba longifolia	6	6	0	20.75	328.42	203.16
Scabiosa mansenensis	7	7	0	0.09	325.14	170.76
Angelica tenuissima	7	5	2	0.12	242.86	126.03
Koelreuteria paniculata	7	6	1	0.29	205.29	90.84
Abelia coreana	7	7	0	0.54	155.19	82.33
Ajuga spectabilis	7	7	0	0.84	212.64	106.92
Lonicera chrysaniha	7	7	0	1.44	361.44	191.80
Patrinia rupestris	7	7	0	10.04	278.37	146.14
Bupleurum falcatum	7	7	0	14.51	225.29	112.60
Aconitum koreanum	7	7	0	20.72	371.73	156.18
Viola variegata	8	7	1	0.10	306.94	85.56
Sedum rotundifolium	8	6	2	0.12	19.04	8.89
Iris ruthenica	8	7	1	0.16	247.68	121.75
Weigela florida	8	8	0	0.41	288.82	77.11
Rhododendron brachycarpum	8	7	1	0.41	112.31	53.47
Sorbus amurensis	8	7	1	0.78	311.14	154.34
Crypsinus hastatus	8	8	0	1.25	203.94	114.40
Pulsatilla koreana	8	8	0	3.31	135.70	60.43
Cirsium chanroenicum	8	8	0	13.78	188.43	103.81
Thuja orientalis	8	8	0	29.16	268.82	153.80
Convallaria keiskei	9	8	1	0.14	210.67	102.46
Thuja koraiensis	9	9	0	3.13	154.51	56.37
Lysimachia coreana	9	9	0	12.81	295.67	144.67
Aconitum trilobum	10	10	0	0.67	126.28	66.04
Hovenia dulcis	10	9	1	0.70	306.92	134.40
Schizopepon bryoniaefolius	10	10	0	2.51	50.72	24.57
Cypripedium macranthum	11	9	2	0.14	269.65	134.25
Buxus microphylla var. koreana	11	10	1	0.60	228.46	75.85
Eranthis stellata	11	11	0	0.89	264.00	140.67
Acer barbinerve	11	11	0	1.77	160.35	74.98
Celtis choseniana	12	11	1	0.10	225.45	112.99
Rhododendron tschonoskii	12	11	1	0.53	82.45	36.04
Taxus cuspidata	12	12	0	0.57	235.58	70.87
Equisetum hyemale	12	12	0	1.04	110.82	37.81
Paulownia coreana	12	12	0	5.92	326.94	158.12
Epimedium koreanum	13	13	0	0.30	181.51	70.12
Symplocarpus renifolius	13	13	0	1.09	298.49	146.93
Prunus davidiana	13	13	0	19.59	345.49	162.94
Lonicera subsessilis	14	14	0	0.80	350.81	145.83
Sorbus commixta	14	14	0	3.98	323.95	110.94
Angelica gigas	15	15	0	1.18	251.32	100.64
Saxifraga punctata	15	15	0	1.72	288.54	154.30
Smilacina bicolor	16	12	4	0.10	321.63	134.36
Oplopanax elatus	16	15	1	0.25	313.33	129.15
Paeonia obovata	17	15	2	0.10	289.52	136.01

Symplocarpus nipponicus	*17*	*15*	*2*	*0.15*	*328.54*	*94.50*
Acer ukurunduense	*17*	*17*	*0*	*0.44*	*145.65*	*54.77*
Euonymus pauciflorus	*17*	*17*	*0*	*8.48*	*349.54*	*159.59*
Spiraea salicifolia	*18*	*18*	*0*	*0.10*	*282.48*	*111.74*
Disporum ovale	*18*	*17*	*1*	*0.52*	*315.76*	*132.28*
Tricyrtis dilatata	*18*	*17*	*1*	*0.54*	*337.89*	*131.97*
Acanthopanax chiisanensis	*18*	*17*	*1*	*0.63*	*296.57*	*147.94*
Acer tegmentosum	*19*	*19*	*0*	*0.22*	*214.61*	*73.93*
Filipendula glaberrima	*19*	*19*	*0*	*2.90*	*248.93*	*103.61*
Lilium cernum	*20*	*18*	*2*	*0.11*	*293.33*	*105.34*
Anemone koraiensis	*20*	*18*	*2*	*0.13*	*258.45*	*89.60*
Nymphoides peltata	*20*	*13*	*7*	*0.20*	*19.02*	*5.56*
Hanabusaya asiatica	*20*	*20*	*0*	*0.38*	*115.49*	*50.62*
Trillium kamtschaticum	*21*	*20*	*1*	*0.21*	*174.04*	*62.47*
Aristolochia contorta	*21*	*21*	*0*	*1.66*	*291.16*	*132.83*
Rhododendron micranthum	*22*	*21*	*1*	*0.13*	*118.49*	*53.64*
Corylopsis coreana	*23*	*19*	*4*	*0.13*	*397.43*	*69.55*
Stewartia koreana	*23*	*21*	*2*	*0.23*	*161.50*	*62.56*
Iris odaesanensis	*24*	*22*	*2*	*0.26*	*285.80*	*89.85*
Scopolia japonica	*25*	*21*	*4*	*0.09*	*320.21*	*135.89*
Phellodendron amurense	*25*	*22*	*3*	*0.18*	*261.68*	*104.59*
Patrina saniculaefolia	*26*	*26*	*0*	*0.35*	*384.06*	*135.12*
Aristolochia manshuriensis	*28*	*24*	*4*	*0.05*	*211.39*	*87.61*
Berchemia berchemiaefolia	*29*	*17*	*12*	*0.06*	*127.16*	*15.34*
Ilex macropoda	*29*	*23*	*6*	*0.11*	*278.57*	*102.20*
Lilium distichum	*30*	*29*	*1*	*0.23*	*371.35*	*152.07*
Syringa wolfi	*31*	*30*	*1*	*0.11*	*340.76*	*100.68*
Anemone reflexa	*31*	*31*	*0*	*0.45*	*245.55*	*103.39*
Prunus yedoensis	*32*	*16*	*16*	*0.07*	*316.12*	*64.65*
Hylomecon hylomeconoides	*35*	*27*	*8*	*0.08*	*89.69*	*31.50*
Gastrodia elata	*37*	*36*	*1*	*0.16*	*397.26*	*149.77*
Viola diamantica	*37*	*37*	*0*	*0.73*	*255.42*	*83.73*
Dicentra spectabilis	*39*	*33*	*6*	*0.11*	*266.04*	*126.55*
Codonopsis lanceolata	*49*	*42*	*7*	*0.08*	*338.26*	*116.02*
Rodgersia podophylla	*59*	*55*	*4*	*0.09*	*333.02*	*102.99*
Cimicifuga heracleifolia	*65*	*59*	*6*	*0.08*	*411.56*	*138.64*
Kalopanax pictus	*69*	*66*	*3*	*0.13*	*427.44*	*146.24*
